# A Nonredundant Phosphopantetheinyl Transferase, PptA, Is a Novel Antifungal Target That Directs Secondary Metabolite, Siderophore, and Lysine Biosynthesis in *Aspergillus fumigatus* and Is Critical for Pathogenicity

**DOI:** 10.1128/mBio.01504-16

**Published:** 2017-07-18

**Authors:** Anna Johns, Daniel H. Scharf, Fabio Gsaller, Hella Schmidt, Thorsten Heinekamp, Maria Straßburger, Jason D. Oliver, Mike Birch, Nicola Beckmann, Katharine S. Dobb, Jane Gilsenan, Bharatkumar Rash, Elaine Bignell, Axel A. Brakhage, Michael J. Bromley

**Affiliations:** a Manchester Fungal Infection Group, Institute of Inflammation and Repair, University of Manchester, Manchester, United Kingdom; b Department of Molecular and Applied Microbiology, Leibniz Institute for Natural Product Research and Infection Biology, Jena, Germany; c Department of Microbiology and Molecular Biology, Institute of Microbiology, Friedrich Schiller University, Jena, Germany; d Transfer Group Anti-Infectives, Leibniz Institute for Natural Product Research and Infection Biology, Jena, Germany; e F2G Ltd., Eccles, Manchester, United Kingdom; Washington University School of Medicine; Washington University School of Medicine

**Keywords:** *Aspergillus fumigatus*, antifungal agents, drug targets, gliotoxin, secondary metabolism, virulence determinants

## Abstract

Secondary metabolites are key mediators of virulence for many pathogens. *Aspergillus fumigatus* produces a vast array of these bioactive molecules, the biosynthesis of which is catalyzed by nonribosomal peptide synthetases (NRPSs) or polyketide synthases (PKSs). Both NRPSs and PKSs harbor carrier domains that are primed for acceptance of secondary metabolic building blocks by a phosphopantetheinyl transferase (P-pant). The *A. fumigatus* P-pant PptA has been shown to prime the putative NRPS Pes1 *in vitro* and has an independent role in lysine biosynthesis; however, its role in global secondary metabolism and its impact on virulence has not been described. Here, we demonstrate that PptA has a nonredundant role in the generation of the vast majority of detectable secondary metabolites in *A. fumigatus*, including the immunomodulator gliotoxin, the siderophores triacetylfusarinine C (TAFC) and ferricrocin (FC), and dihydroxy naphthalene (DHN)-melanin. We show that both the lysine and iron requirements of a *pptA* null strain exceed those freely available in mammalian tissues and that loss of PptA renders *A. fumigatus* avirulent in both insect and murine infection models. Since PptA lacks similarity to its mammalian orthologue, we assert that the combined role of this enzyme in both primary and secondary metabolism, encompassing multiple virulence determinants makes it a very promising antifungal drug target candidate. We further exemplify this point with a high-throughput fluorescence polarization assay that we developed to identify chemical inhibitors of PptA function that have antifungal activity.

## IMPORTANCE

Fungal diseases are estimated to kill between 1.5 and 2 million people each year, which exceeds the global mortality estimates for either tuberculosis or malaria. Only four classes of antifungal agents are available to treat invasive fungal infections, and all suffer pharmacological shortcomings, including toxicity, drug-drug interactions, and poor bioavailability. There is an urgent need to develop a new class of drugs that operate via a novel mechanism of action. We have identified a potential drug target, PptA, in the fungal pathogen *Aspergillus fumigatus*. PptA is required to synthesize the immunotoxic compound gliotoxin, DHN-melanin, which *A. fumigatus* employs to evade detection by host cells, the amino acid lysine, and the siderophores TAFC and FC, which *A. fumigatus* uses to scavenge iron. We show that strains lacking the PptA enzyme are unable to establish an infection, and we present a method which we use to identify novel antifungal drugs that inactivate PptA.

## INTRODUCTION

Humans are constantly challenged by the threat of fungal infection. Estimates put the number of individuals who acquire superficial fungal infections at about 1.7 billion (1). There is also a significant proportion of invasive fungal infections which are difficult to treat and lead to an estimated 1.5 million deaths each year (2). The incidence of fungal disease increased significantly in the latter part of the 20th century, and this has been attributed to the expansion of immune-deficient populations, particularly those who receive immunosuppressive therapies (3). *Aspergillus fumigatus* is a filamentous fungal pathogen and is the leading cause of invasive aspergillosis, a fungal disease that causes more than 200,000 life-threatening infections annually, with mortality rates of up to 95%, and a range of chronic diseases that cause significant morbidity and mortality (2). The problems associated with the medications used to treat fungal disease, such as adverse side effects in patients, drug-drug interactions, and increasing antifungal resistance (4), are significant. These problems, coupled with the limited number of agents currently in development (5), highlight the need to identify new classes of antifungals directed against novel drug targets.

The success of many pathogens, including *A. fumigatus*, is directly linked to their ability to produce potent and active secondary metabolites, both as part of their defense system and for adaptation to host environments (6). For instance, siderophore-mediated iron sequestration and uptake are required by *A. fumigatus* to establish an infection in iron-limited environments, such as murine lungs (7). Dihydroxy naphthalene (DHN)-melanin plays a multifactorial role in virulence by quenching oxygen radicals, inhibiting acidification of phagolysosomes, and masking fungal pattern-associated molecular patterns (PAMPs), leading to reduced detection and killing by the host defense system (8–12). Gliotoxin has been associated with suppression of the adaptive immune response, reduction of polymorphonuclear leukocyte-mediated inflammation, and the prevention of the respiratory burst in human polymorphonuclear leukocytes (13–15).

The production of the aforementioned secondary metabolites, and over 200 others (16), is governed by the action of a plethora of nonribosomal peptide synthetase (NRPS) and polyketide synthase (PKS) enzymes. These enzymes are typically organized in modules, with each module being responsible for the incorporation of a substrate to a peptide chain or polyketide. An essential component of each module is a carrier domain to which intermediates of the biosynthetic pathway are attached. Carrier domains need to be primed with a cofactor to receive pathway intermediates. The priming function is provided by 4′-phosphopantetheinyl transferases (4′-PPTases), which transfer and covalently tether the cofactor 4′-phosphopantetheine (P-pant) from coenzyme A (CoA) to a conserved serine residue within the carrier domain (17, 18) (see Fig. S1 in the supplemental material).

10.1128/mBio.01504-16.1 FIG S1  Phosphopantetheinylation. The 4′-phosphopantetheine (P-pant) group within coenzyme A is transferred to a conserved serine residue in a peptidyl carrier domain of an inactive apo-carrier protein to create an active holo-carrier protein. This process is facilitated by 4′-phosphopantetheinyl transferase (4′-PPTase) (adapted from reference 6). Download FIG S1, DOCX file, 0.04 MB. Copyright © 2017 Johns et al. 2017 Johns et al. This content is distributed under the terms of the Creative Commons Attribution 4.0 International license.

The majority of filamentous fungi have distinct, functionally diverse, and nonredundant PPTases that can be categorized into 3 families (19). Type I enzymes, typified by the 120-amino-acid (aa) bacterial acyl carrier protein synthase from which the family derives its name (ACPs type), act to prime acyl carrier proteins (ACPs) involved in type II (mitochondrial) fatty acid synthesis. Type III enzymes exist as integral domains of the C terminus of type I fatty acid synthases (FASs) (20). Our key focus in this study relates to family II, Sfp-type enzymes. Named after a protein first identified as being required for the biosynthesis of the NRPS-derived antimicrobial peptide surfactin, Sfp-type enzymes are thought to have evolved due to a gene duplication of the ACPs-type PPTases and, in contrast to the ACPs type, which typically activate a single enzyme, are able to phosphopantetheinylate a broader range of target proteins (19).

In *Aspergillus nidulans*, the Sfp-type 4′-PPTase gene is known as *npgA* (21). NpgA has been well characterized, shows a wide array of activity (22), and is responsible for the phosphopantetheinylation of enzymes involved in primary and secondary metabolism, including the polyketide synthase WA, which is required for the first committed step in melanin pigment biosynthesis (23), α-aminoadipate reductase (AarA), which is required for lysine biosynthesis, the NRPSs SidC and SidD, components of intracellular and extracellular siderophore biosynthesis, and the NRPS δ-(l-α-aminoadipyl)-l-cysteinyl-d-valine synthetase, which is required for penicillin biosynthesis (22, 24, 25). Without supplementation with the siderophore triacetylfusarinine C (TAFC) and lysine, an *npgA* null mutant is inviable; however, when supplemented, the null mutant grows and generates characteristic albino spores, consistent with the loss of melanin biosynthesis (22). The *A. fumigatus* orthologue of NpgA is PptA and, as in *A. nidulans*, it has been shown to be involved in lysine biosynthesis, as a *pptA* null mutant is auxotrophic for lysine and PptA was able to activate AarA in an *in vitro* assay (26). Consistent with a role for PptA in activation of the WA orthologue PksP, *pptA* null mutants also have albino conidia. In contrast to the situation in *A. nidulans*, however, *A. fumigatus* is able to acquire free iron without the need for siderophores, due to the action of an operative reductive iron assimilatory system (7); therefore, *pptA* null mutants do not need to be supplemented with TAFC in iron-replete environments (15).

The involvement of PptA in the production of many virulence factors linked to both primary (lysine) (22, 27, 28) and secondary metabolism (29, 30) suggests it is likely to play a prominent role in pathogenicity in mammalian hosts and hence points to its possible value as a novel antifungal drug target, as previously proposed by Márquez-Fernández et al. (31). Here, we show that PptA provides a critical metabolic hub that has an essential role in the production of secondary metabolites, growth under iron-limiting conditions, and virulence of *A. fumigatus*. We further demonstrate the suitability of this enzyme as an antifungal target by using a high-throughput screening (HTS) enzymatic assay to identify inhibitors of PptA.

## RESULTS

### The nutritional requirements of an *A. fumigatus pptA* null mutant exceed the nutrients scavangable within a mammalian host environment.

**FIG 1:**
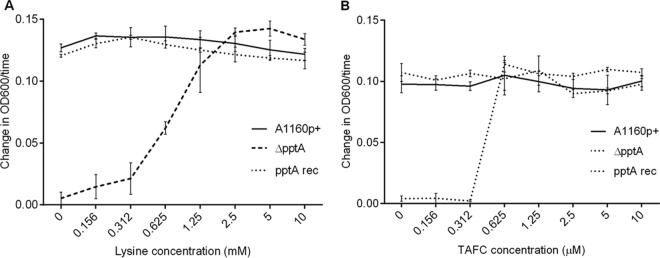
Dose response of a *pptA* null isolate to increasing concentrations of TAFC and lysine**.** (A) Response in RPMI medium supplemented with TAFC (10 µM) and an increasing concentration of lysine. (B) Response in RPMI medium supplemented with lysine (10 mM) and an increasing concentration of TAFC.

Previous studies have shown that *A. fumigatus* strains lacking *pptA* require supplementation with both lysine and iron to sustain growth and form nonpigmented conidia. To assess in the *pptA* null mutant whether lysine and iron requirements exceed those available in a mammalian host environment, the minimum requirement of this strain for both lysine and the extracellular siderophore TAFC, as a source of free iron, was determined (Fig. 1). At least 1.25 mM lysine was required to restore the growth rate of the *pptA* null mutant to wild-type levels; however, growth was evident (approximately 10% compared to the parental strain) at 0.16 mM. It is noteworthy that levels of the free amino acid in human plasma can reach up to 0.28 mM (32, 33). The minimal requirement of the *pptA* null mutant for TAFC was 6.3 × 10^−4^ mM. This is significantly higher than free iron levels found in humans, where the iron is under strict homeostasis and is mostly found tightly bound to hemoglobin, transferrin, lactoferrin, and ferritin. It is estimated that the concentrations of free iron in human secretions and serum are approximately 10^−15^ mM and 10^−21^ mM, respectively (34, 35). If this information were taken in isolation, it would suggest that iron limitation in host tissues would prevent a strain lacking PptA activity from growing in a human host.

### Immunotoxic secondary metabolites are absent from culture supernatants of a *pptA* null mutant.

**FIG 2:**
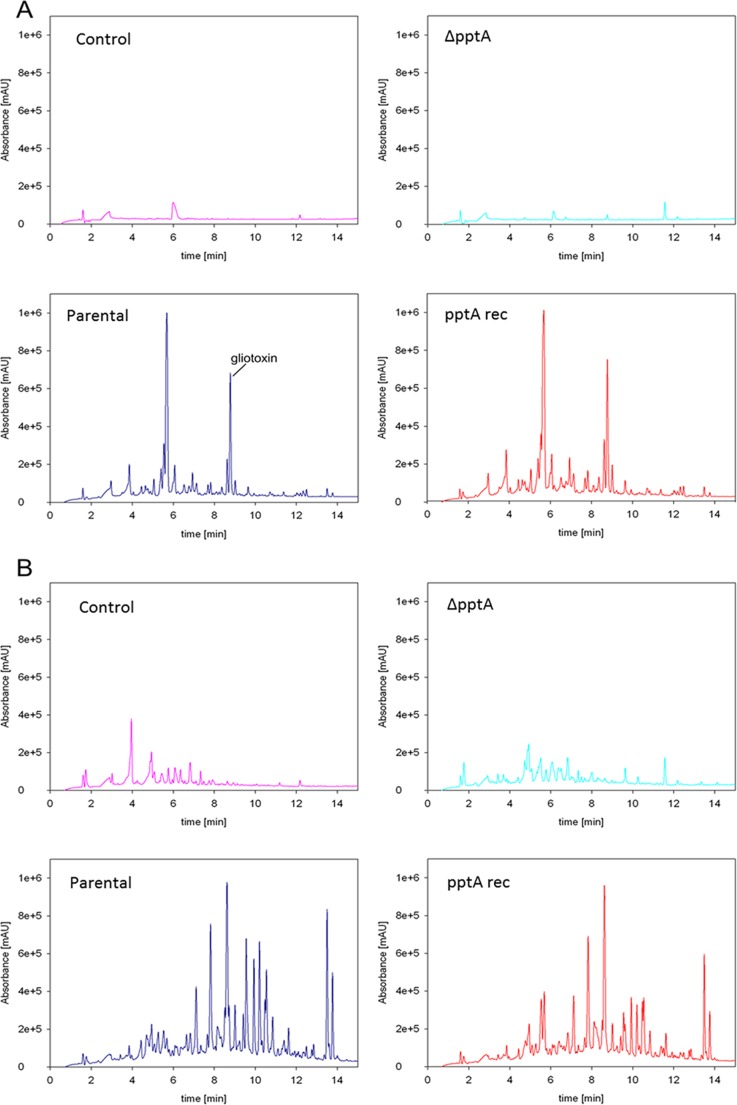
Analysis of secondary metabolite production by LC-MS. Secondary metabolite profiles from a medium-only sample (control) alongside extracts from culture supernatants from the isogenic control (parental), the *pptA* null mutant (Δ*pptA*), and a reconstituted isolate (*pptA* rec). (A) Seven-day-old culture supernatants isolated from Czapek-Dox medium; the parental strain and reconstituted isolate produced gliotoxin (marked) as well as other metabolites, whereas the *pptA* null mutant did not. (B) Seven-day-old culture supernatants isolated from Sabouraud medium; the parental strain and reconstituted isolate produced fumigaclavine C (retention time, 8.20 min), fumiquinazole A (retention time, 9.56 min), fumiquinazoline C (retention time, 9.9 min), pyripyroprene A (retention time, 11.28 min), fumagillin (retention time, 13.50 min), and traces of triacetylfusarinine C (detectable as a mass peak, by MS analysis only) among other metabolites. The *pptA* null mutant showed a trace similar to that of the medium “control”; however, unidentified peaks with retention times of 9.5 and 11.5 min were seen.

Márquez-Fernández et al. in 2007 (31) defined a role for CfwA/NpgA in the production of NRPS- and PKS-derived secondary metabolites in *A. nidulans*. As secondary metabolites are thought to play a key role in fungal virulence (36–38), we assessed if this role is conserved in *A. fumigatus*. The capability of the Δ*pptA* strain to generate secondary metabolites was assessed by analyzing liquid chromatography-mass spectrometry (LC-MS) absorbance spectra from 7-day-old culture supernatants. Several non-medium-derived peaks, including those corresponding to the major metabolites gliotoxin, fumigaclavine C, fumiquinazole A, fumiquinazoline C, pyripyroprene A, and fumagillin, were observed in extracts from the parental strain grown on defined medium (Czapek-dox) (Fig. 2A) and complex medium (Sabouraud [SAB]) (Fig. 2B). Additionally, mass peaks for TAFC were identified in supernatants derived from the parental isolate (Table S1). None of these peaks was identified in supernatant extracts from the *pptA* null isolate, which showed an apparent lack of secondary metabolite production. The metabolic profile of the *pptA* null isolate resembled the medium-only control profile under tested conditions, with the exception of two prominent peaks with retention times of 9.5 and 11.5 min. The identities of these peaks were not investigated. The metabolite profile of the reconstituted isolate was consistent with that of the parental isolate.

10.1128/mBio.01504-16.5 TABLE S1  Predicted and detected masses of TAFC identified in culture supernatants from the isogenic parent (wt) and the *pptA* reconstituted strain pptArec. Download TABLE S1, DOCX file, 0.01 MB. Copyright © 2017 Johns et al. 2017 Johns et al. This content is distributed under the terms of the Creative Commons Attribution 4.0 International license.

### The *pptA* null mutant stimulates dendritic cells to produce immunomodulatory cytokines and enhances phagolysosome acidification.

**FIG 3:**
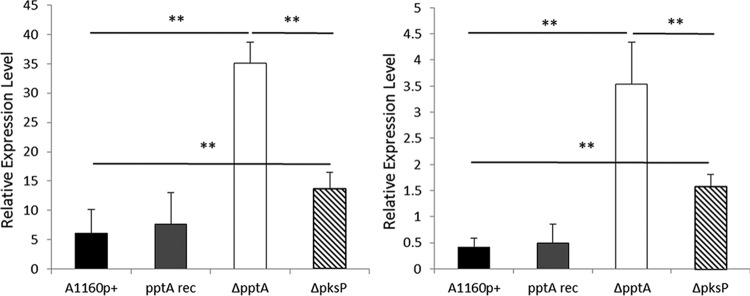
Response of human dendritic cells to *pptA* and *pksP* null mutants**.** Dendritic cells were cultured with paraformaldehyde-fixed conidia for 24 h. Total RNA was extracted, and the relative expression levels of IL-1β (A) and IL-6 (B) were assessed by QRT-PCR. Data represent the mean results of 3 replicates, and error bars signify the standard deviations. **, *P*  < 0.01.

Previous studies have demonstrated that DHN-melanin masks the recognition of pathogen-associated molecular patterns on the conidia of *A. fumigatus* and reduces phagolysosome acidification (10, 12). Furthermore, loss of DHN-melanin biosynthesis in *pksP* mutants of *A. fumigatus* leads to the development of an amorphous and hydrophilic coating of protein around conidia, which coincides with enhanced detection by the immunological sentinel dendritic cells (DCs), leading to release of a number of immunomodulatory cytokines (10–12, 39). As the Δ*pptA* mutant isolate lacks the ability to produce DHN-melanin, we hypothesized that it would be detected more readily by human DCs than an isogenic control isolate (parental strain A1160p+). We generated an isogenic *pksP* null strain, Δ*pksP*, as a nonpigmented, *pptA*-expressing control for this study. Fixed spores of the parental A1160P+ strain, Δ*pptA*, the *pptA* reconstituted isolate, and Δ*pksP* were used to challenge human dendritic cells. An increase in relative expression of the proinflammatory cytokines interleukin-1β (IL-1β) and IL-6 in the DCs was observed when they were challenged with either the Δ*pptA* or Δ*pksP* strains (*P*  < 0.01) compared to the response from the parental strain. Interestingly, the level of response to the *pptA* null isolate was significantly (*P* < 0.01) and reproducibly (*n* = 2 independent experiments) greater than the response to the Δ*pksP* mutant (Fig. 3).

**FIG 4:**
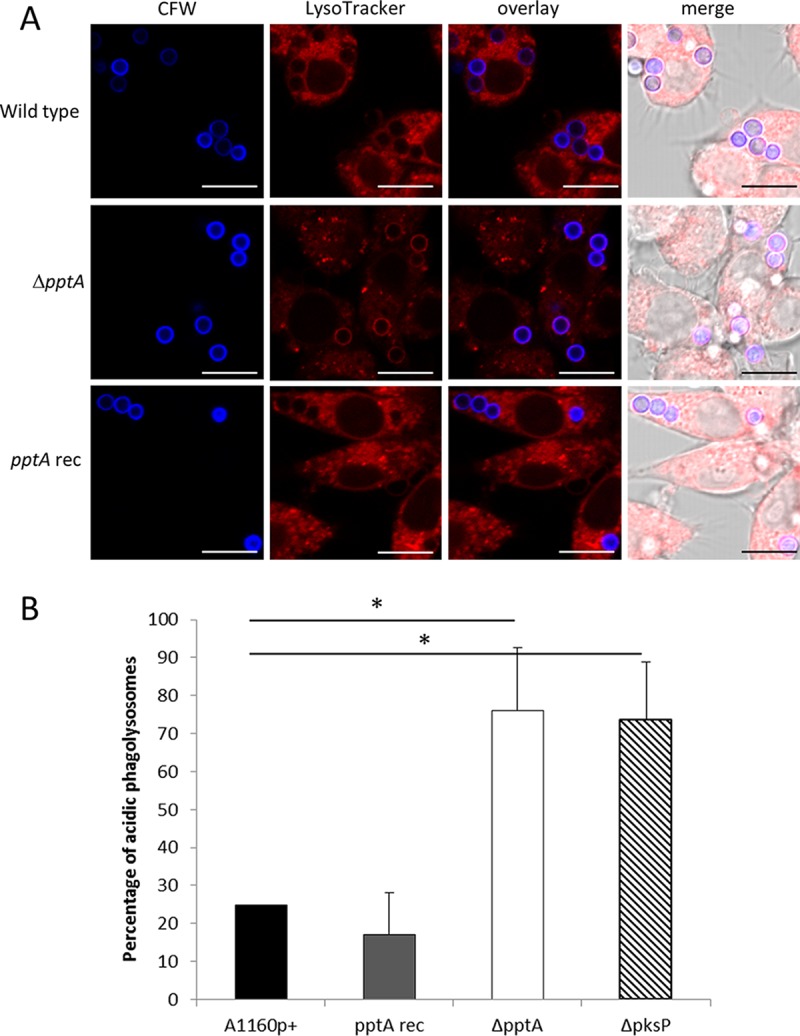
Detection of conidia in acidified compartments after phagocytosis by RAW264.7 macrophages. (A) Calcofluor white (CFW)-labeled conidia were intracellularly colocalized by using LysoTracker Red-DND99 in RAW 264.7 macrophages, and the ratio of acidified phagolysosomes was determined after 2 h of coincubation with an MOI of 2. (B) The percentages of conidia in acidic phagolysosomes. Data represent mean results and SDs (error bars) of three independent experiments.

*A. fumigatus* conidia and melanin ghosts can inhibit the acidification of phagolysosomes of alveolar macrophages, monocyte-derived macrophages, and human neutrophil granulocytes, whereas strains lacking DHN-melanin cannot inhibit acidification (10, 40). To assess if a similar phenomenon occurred with the nonpigmented Δ*pptA* mutant isolate, acidification of phagolysosomes was measured using LysoTracker, a dye which shows red fluorescence in an acidic compartment (Fig. 4). Seventy-six percent of the mutant Δ*pptA* conidia were found residing in acidified compartments, compared to 25% of the parental strain, A1160p+. The level of increase in acidification was similar between strains Δ*pptA* (76%) and Δ*pksP* (74%), which suggests DHN-melanin as the main factor in prevention of phagolysosomal acidification in the *pptA* null mutant.

### PptA is vital for virulence of *A. fumigatus.*

**FIG 5:**
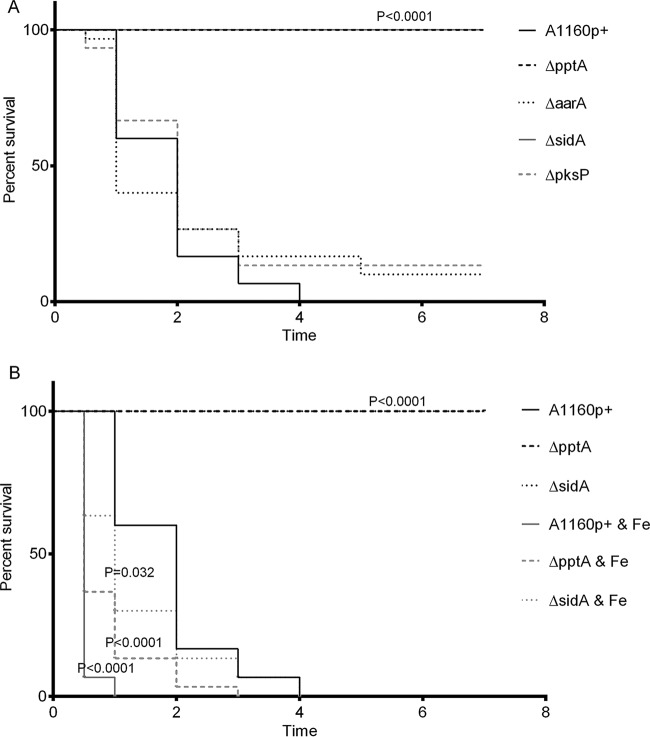
The effects of a series of null mutants of *A. fumigatus* on the mortality of larvae (A) and the effects of iron treatment on the pathogenicity of Δ*pptA* and Δ*sidA* mutants in a larvae model (B). (A) Larvae (*n* = 30 per group) were infected with 1 × 10^4^ spores on day 0 from the indicated strains on day 0. The percent survival was plotted on a Kaplan-Meier survival curve. (B) Larvae (*n* = 30 per group) were infected with 1 × 10^4^ spores from the indicated strains on day 0. Half of the larvae from each group were given a daily treatment of 1.5 mM FeSO_4_. The percent survival was plotted on a Kaplan-Meier survival curve.

The significant growth defects *in vitro* and lack of secondary metabolite production indicated that the *pptA* null strain may lack the ability to establish an infection. To test this hypothesis, we first assessed the virulence of the Δ*pptA* strain in the *Galleria mellonella* wax moth virulence model (41). The control isolate, A1160p+, caused 100% mortality 4 days postinfection, whereas all larvae in the cohort infected with the *pptA* null mutant survived (Fig. 5A).

To assess the underlying cause of this loss of virulence, the pathogenicity of additional isogenic strains lacking either the ability to produce siderophores (Δ*sidA* strain), lysine (Δ*aarA* strain), or DHN-melanin (Δ*pksP* strain) was assessed. A significant difference in virulence was observed only for the Δ*sidA* strain (*P*  < 0.0001 versus the control isolate) (Fig. 5A). This suggests, at least in the larval model, that siderophore biosynthesis but not biosynthesis of lysine or other secondary metabolites is the primary cause of loss of virulence in the *pptA* mutant. To further confirm this, larvae infected with A1160p+, strain Δ*pptA*, and strain Δ*sidA* were administered with 10 µl of 1.5 mM FeSO_4_ every day for the length of the study. Under these conditions, virulence was increased to a higher level than in the untreated parental strain in both strain Δ*pptA* (*P* < 0.0001) and strain Δ*sidA* (*P* < 0.05). Interestingly, there was also an increase in parental strain virulence when supplemental iron was administered, decreaseing the time at which 100% mortality was achieved from +4 days to +1 day (*P* < 0.001) (Fig. 5B). One hundred percent survival was observed in larvae that received iron supplementation without infection.

**FIG 6:**
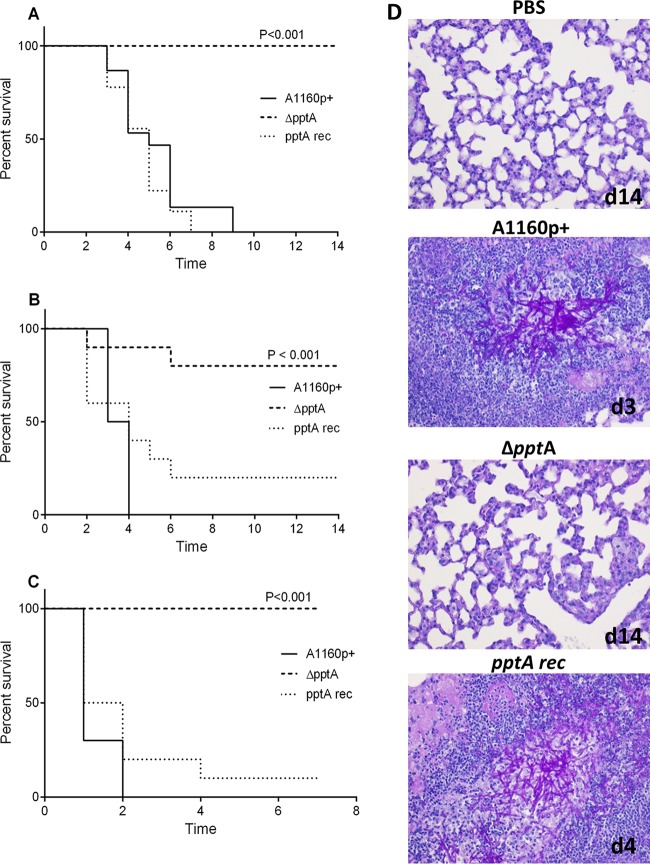
Assessing the effect of loss of *pptA* on virulence in murine infection models**.** Mice were infected with the parental (A1160p+), *pptA* null mutant (Δ*pptA*), or the reconstituted isolate (*pptA* rec). (A to C) Kaplan-Meier plots of results from mice treated via (A) intranasal infection with 2 × 10^4^ spores after mice were rendered neutropenic by treatment with cyclophosphamide, (B) intranasal infection with 2 × 10^5^ spores after cortisone-acetate treatment, or (C) intravenous infection with 1.3 × 10^5^ spores. A log rank analysis was used to compare results after Δ*pptA* infection versus infection with the parental strain (A1160p+). In all cases (A to C), *P* values were less than 0.001. (D) Histopathology of representative sections of lungs from *A. fumigatus*-infected mice from the study shown in panel B. The presence of invasive fungal hyphae (pink), destructed lung tissue, and infiltration of immune cells (purple nuclei) were confirmed in lungs of mice infected with conidia from the parental strain, the *pptA* null isolate, or the reconstituted isolate. A lung section of a PBS sham-infected mouse is shown as a control.

For a more clinically relevant assessment of both pulmonary (Fig. 6A) and disseminated (Fig. 6C) disease, we modeled intranasal and intravenous infections, respectively, using parental or strain Δ*pptA* inocula in leukopenic mice. Regardless of the infection model, all mice infected with the parental strain succumbed to disease and were sacrificed before the end of each study. However, and in keeping with the results from the larval virulence study, 100% survival was seen in the cohorts infected with the Δ*pptA* mutant isolate in both models. The virulence of the reconstituted isolate was indistinguishable from the parental isolate (*P* = 0.3043 and 0.1750 for intranasal and intravenous infections, respectively). Additionally, we carried out a further pulmonary infection using mice treated with cortisone acetate; these mice retain the ability to recruit neutrophils and monocytes (Fig. 6B). Eighty percent of mice infected with strain Δ*pptA* survived until the end of the study, whereas none of the mice in the parental study survived. Histological analysis of the lungs from deceased mice in the mutant Δ*pptA* cohort were devoid of fungal hyphae, indicating that factors other than invasive hyphal growth were responsible for the observed mortality. Histological data from mice that survived to day 14 indicated that the Δ*pptA* mutant had been totally cleared from the lungs (Fig. 6D).

### Type II PPTases are phylogenetically diverse.

Anti-infective drug targets that lack sequence similarity to any human orthologues are preferred, as this implies a reduced potential for drug toxicity. It is also preferable that a target shares high similarity over a range of related pathogens, indicating the potential to develop a drug with a broad spectrum of activity. To evaluate the relatedness of type II PPTases, a phylogram was constructed using the sequences homologous to the *A. fumigatus* PptA from a number of relevant mammalian, bacterial, and fungal proteins.

**FIG 7:**
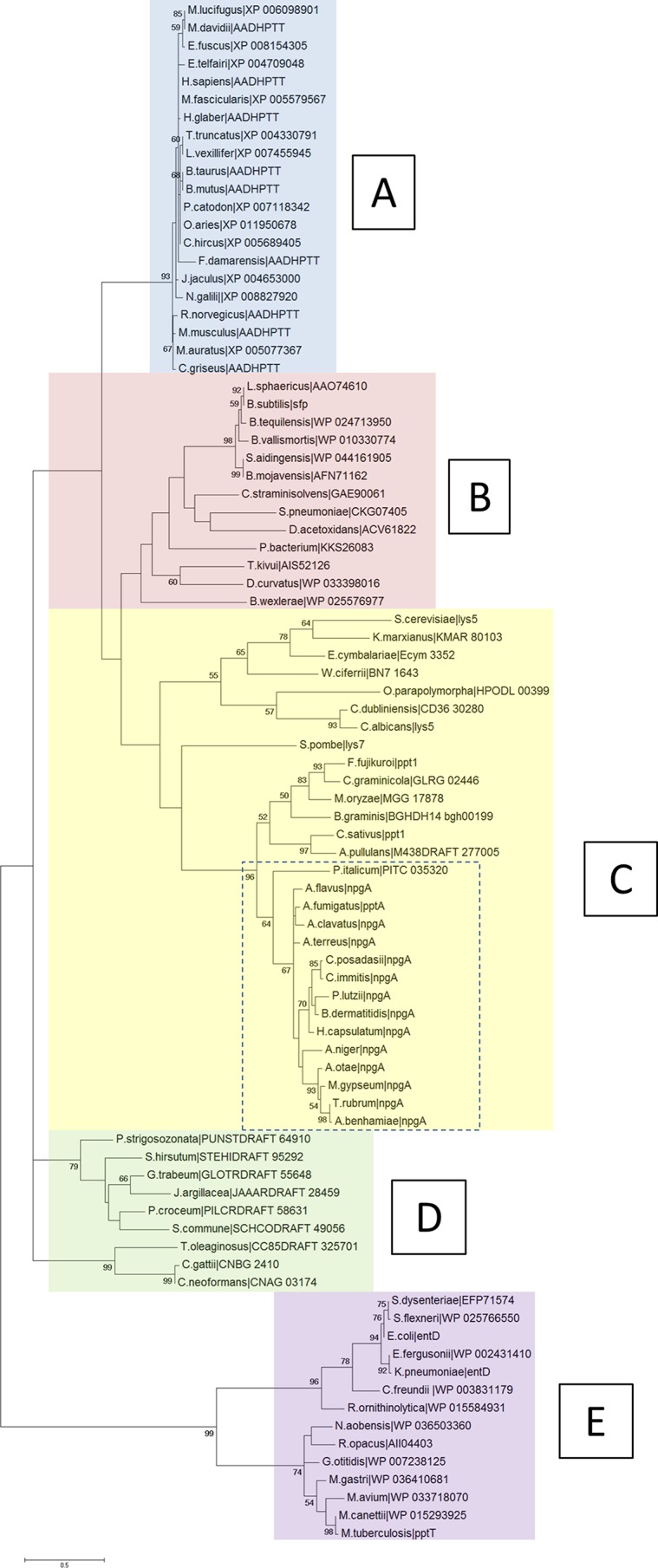
Molecular phylogenetic analysis results for the Sfp-PPTases, based on the maximum likelihood method**.** The evolutionary history was inferred by using the maximum likelihood method based on the JTT matrix-based model. The percentages for trees in which the associated taxa clustered together are shown next to branches. The initial tree(s) for the heuristic search was obtained by applying the neighbor-joining method to a matrix of pairwise distances estimated using a JTT model. The tree is drawn to scale, with branch lengths based on the number of substitutions per site. Evolutionary analyses were conducted using MEGA6.

Five separate clusters were identified (Fig. 7). Cluster A contains the highly conserved mammalian PPTases with very short branch lengths (<0.12 amino acid substitutions per site), while clusters B and E consist of enzymes from *Firmicutes* and *Proteobacteria*/*Actinobacteria*, respectively. Interestingly, of the fungal enzymes analyzed, 2 distinct clades are formed, representing *Ascomycota* (cluster C) and *Basidiomycota* (cluster D). It is particularly evident that the PPTases in the *Ascomycota* group are highly divergent. This was exemplified by further *in silico* analysis; no significant similarity was found when the *A. fumigatus* PptA enzyme was analyzed with BLASTp against the *Candida albicans* genome, and a low identity of 31% (24% coverage) was observed against the *Saccharomyces cerevisiae* PPTase. However, there does appear to be a distinct, relatively well-conserved grouping within this cluster, which includes PPTases from the aspergilli as well as enzymes from other pathogenic fungi, including *Histoplasma capsulatum*, *Arthroderma benhamiae*, *Coccidioides immitis*, and *Coccidioides posadassi* (Fig. 7, dashed box). Proteins found in this cluster have short branch lengths (<0.15 amino acid substitutions per site), alignment scores of >50%, and percent identities ranging from 47 to 72% (Table S2).

10.1128/mBio.01504-16.6 TABLE S2  List of ClustalW pairwise alignment scores and BLASTp analysis results for *A. fumigatus* PptA (NA, no significant similarity found; shading represents ClustalW pairwise alignment scores of >50%). Download TABLE S2, DOCX file, 0.02 MB. Copyright © 2017 Johns et al. 2017 Johns et al. This content is distributed under the terms of the Creative Commons Attribution 4.0 International license.

**FIG 8:**
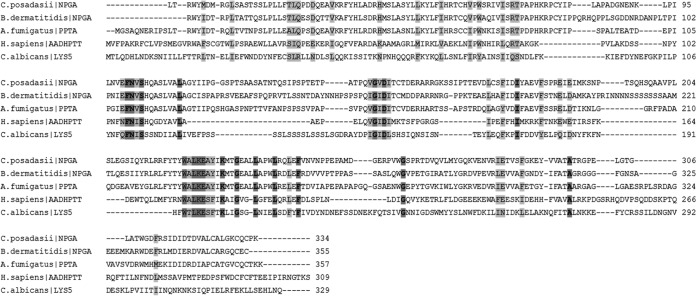
Sequence alignments Sfp-PPTases. Sequence alignments of *A. fumigatus* PptA, the human orthologue AASDHPPT, and fungal pathogen orthologues *Candida albicans* LYS5, *Coccidioides posadassii* NPGA, and *Blastomyces dermatitides* NPGA. Dark gray shading and bold lettering indicate positions which have a single, fully conserved residue. Light gray shading indicates conservation between groups of strongly similar properties, with scores of >0.5 in the Gonnet PAM 250 matrix. Boxes indicate the conserved signature sequence used to distinguish PPTases. Bacterial Sfp-PPTases have a conserved characteristic signature within their sequence: (I/V/L)G(I/V/L/T)D(I/V/L/A)(x)n(F/W)(A/S/T/C)xKE(S/A)h(h/S)K(A/G), where x’s represent chemically disparate amino acids, n represents 38 to 41 aa, and h is an amino acid with a hydrophobic side chain (68). Interestingly, we found the signature of the fungal Sfp-PPTases analyzed in this study diverged somewhat from the conserved sequence: (I/V/L)G(I/V/A/T)D(I/V/L)(x)n(F/W)(A/S/T/C)x(K/R)E_(S/A)_h(h/S)K(M/L/A/F), where n is 41 to 107 aa (Fig. S2).

The human and *A. fumigatus* proteins share an ancestor; however, they have diverged into 2 separate clusters (A and C, respectively). *In silico* analysis showed that the PptA protein shared only 26% identity (61% coverage) and had a low ClustalW pairwise alignment score of 21% with the human orthologue, l-aminoadipate-semialdehyde dehydrogenase–phosphopantetheinyl transferase (AASDHPPT) (http://blast.ncbi.nlm.nih.gov/) (Fig. 8). If a low percent identity can be considered a barometer to indicate target selectivity, PptA compares favorably when placed in context with the target of the azole class antifungal drug Cyp51A, which shares 38% identity (92% coverage) and a ClustalW pairwise alignment score of 35% for human lanosterol 14-alpha demethylase.

10.1128/mBio.01504-16.2 FIG S2  Fungal Sfp-PPTase signatures. Fungal Sfp-PPTases can be identified based on the modified signature (I/V/L)G(I/V/A/T)D(I/V/L)**(**x**)**n(F/W)(A/S/T/C)x(K/R)E_(S/A)_h(h/S)K(M/L/A/F), where n is 41 to 107 aa. Gray shading indicates a conserved signature. Download FIG S2, DOCX file, 0.2 MB. Copyright © 2017 Johns et al. 2017 Johns et al. This content is distributed under the terms of the Creative Commons Attribution 4.0 International license.

### A fluorescence polarization-based assay defines the PptA mode of action and is amenable to high-throughput screening for molecular inhibitors.

**FIG 9:**
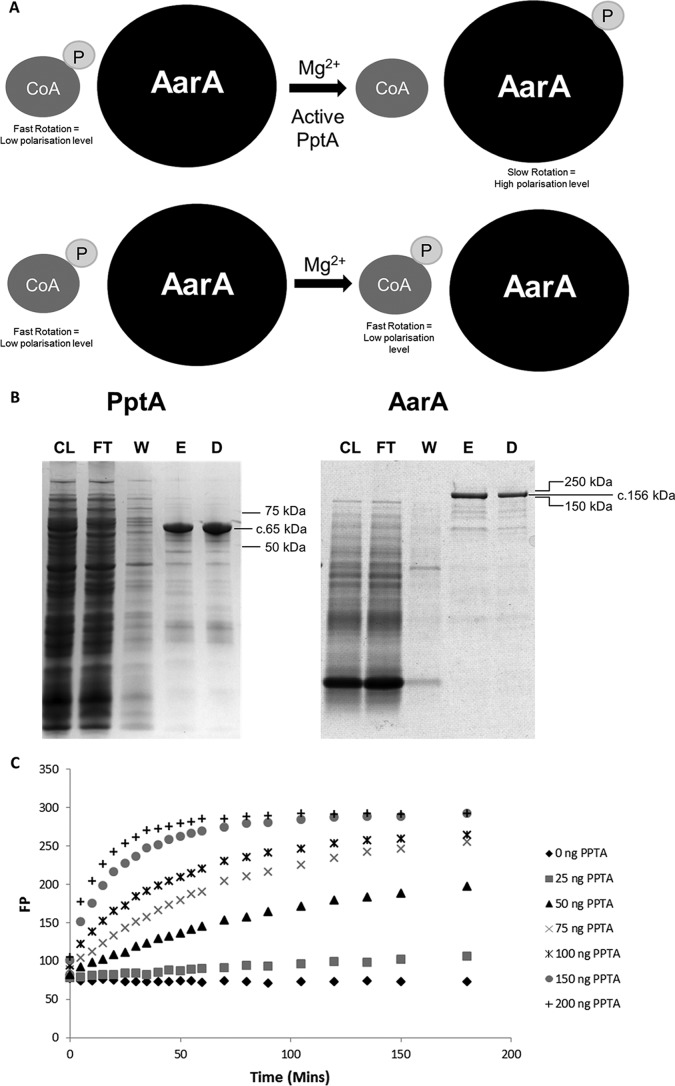
(A) Schematic representation of results of the FP assay**.** Fluorescent polarization is a method of differentiating molecules based on their rotational speed. Nominally, small molecules rotate faster than large molecules. If a fluorophore is attached to a small, quickly rotating molecule (such as CoA) and is excited by polarized light, the light emitted is depolarized. This is in contrast to a large, slowly rotating molecule (such as AarA), which will emit polarized light. (B) PptA-GST and AarA-pManHis protein expression and purification**.** *E. coli* BL21(DE3) cells were transformed with pGEX6P1_PptA and pManHis_AarA, grown to an optical density of >0.5, and then incubated in the presence or absence of 0.5 mM isopropyl-β-d-thiogalactopyranoside (IPTG) for 18 h at 18°C. PptA and AarA were purified from the bacterial lysate by glutathione-Sepharose resin affinity chromatography and Ni-NTA His bind resin affinity chromatography, respectively. Protein fractions were separated by polyacrylamide gel electrophoresis on Mini-Protean TGX stain-free gels (Life Technologies, Inc.) followed by staining with Instant Blue reagent (Expedeon). The molecular masses (in kilodaltons) from a Precision Plus protein ladder are marked. Lane CL, *E. coli* cell lysate following IPTG induction from supernatant; lane FT, flowthrough from the affinity chromatography; lane W, wash to remove unbound proteins; lane E, PptA and AarA purified by affinity chromatography; lane D, PptA and AarA proteins desalted via Sephadex G-25 (PD10 columns; GE Healthcare Life Sciences). PptA-GST and AarA-pManHis showed strong bands at ca. 65 kDa and ca. 156 kDa, respectively. (C) Kinetic evaluation of the titration of PptA (0 to 200 ng per reaction mixture) in the fluorescence polarization assay.

The activity of *A. fumigatus* PptA (AFPptA) has previously been assessed by monitoring the transfer of a fluorescently labeled phosphopantetheinyl group from CoA in a gel shift assay (26); however, this is unsuitable for high-throughput screening of chemical inhibitors. We therefore assessed an alternative strategy, using fluorescence polarization (FP) to monitor the transfer of the labeled phosphopantetheinyl group to an acceptor protein (Fig. 9 shows a schematic representation of the assay).

Attempts to isolate an N-terminal His-tagged derivative of PptA were hampered because the purified protein had limited solubility. We therefore decided to generate an N-terminal glutathione *S*-transferase (GST)-tagged derivative with the aim to increase solubility and stability. Purified, soluble AFPptA-GST was isolated from *Escherichia coli* BL21(DE3) cells and had an estimated mass of 65 kDa, which correlates well with the predicted mass for the fusion protein (65.5 kDa) (Fig. 9B). The cognate acceptor protein AFAarA was expressed in *E. coli* (Fig. 9B) and purified with an estimated mass of 150 kDa, which again correlated with the predicted mass of 156 kDa.

A fluorescently labeled CoA substrate was generated using the thiol-reactive fluorophore BODIPY iodoacetamide. To ensure that the PptA-GST fusion protein was functional, we confirmed its ability to transfer the P-pant group from BODIPY CoA substrate in the aforementioned gel shift assay (15). Fluorescence polarization assays were performed in 50-μl reaction volumes under previously defined buffer conditions (15) in 384-well plates. The amount of carrier protein was fixed at 750 ng per reaction mixture, and the reaction was stopped by addition of EDTA to a final concentration of 60 mM. Optimal enzyme concentrations were determined by kinetic experiments with concentrations of enzyme ranging from 25 ng to 200 ng per reaction mixture at ambient temperature (Fig. 9C). We considered several factors when assessing the assay, including obtaining a near-linear relationship between enzyme activity and time, limiting the time period of the assay so as to increase throughput while providing sufficient time to allow the assays to be conducted in sensible batch sizes, and providing a sufficient signal-to-noise ratio. Optimal conditions where FP was maximized while retaining a linear increase in FP over a 30-min period were achieved with 100 ng PptA (Fig. 9C).

The quality and consistency of the assay were assessed by calculating the Z′ factor, a statistical parameter used to assess signal dynamic range and assay variability (42). Mean maximal and background signal values were obtained from 384 replicates of the assay performed in the absence and presence of the inhibitor EDTA (60 mM), respectively. These data indicated that the assay was suitable for high-throughput screening, giving a Z′ value of 0.76, which is well above the minimum value of 0.5 used to define an acceptable HTS (Fig. S3). Another important logistical factor of high-throughput screens is the length of time between stopping a reaction and reading the signal. If reactions are stable over long periods of time, larger assay batches can be processed and the need to synchronize the performing and reading of an assay result diminishes, reducing overall costs. We therefore assessed the stability of the assay signal over time. The assay was remarkably stable, as the mean FP maximal (FP_max_) millipolarization (mP) values taken immediately after addition of stop solution were 205 (*n* = 380; coefficient of variation [CV], 3.7); even after 1 week, this value remained unchanged for the same samples at an FP_max_ of 205 mP (*n* = 380; CV, 2.7). The high Z' value, along with the stability and simplicity of the assay, make it suitable for HTS applications.

10.1128/mBio.01504-16.3 FIG S3  Repeatability determination of AFPptA assay results in a 384-well plate. Each point represents the FP value obtained from the AFpptA assay in the presence (black) or absence (gray) of the metal chelator and assay inhibitor EDTA. Download FIG S3, DOCX file, 0.03 MB. Copyright © 2017 Johns et al. 2017 Johns et al. This content is distributed under the terms of the Creative Commons Attribution 4.0 International license.

### PptA is a druggable target in *Aspergillus* species.

**FIG 10:**
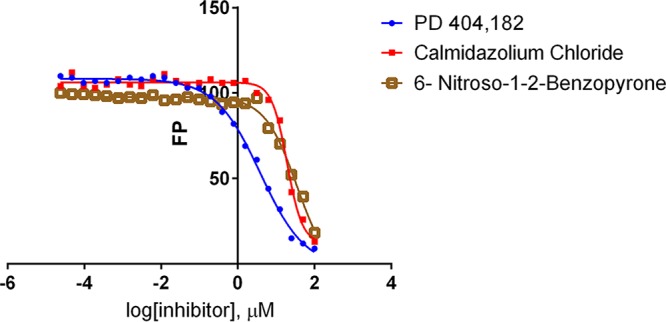
Inhibition of *A. fumigatus* PPTA with PD 404,182, 6-nitroso-1,2-benzopyrone, and calmidazolium chloride. The calculated IC_50_s were 3.9, 35.2, and 19.2 µM, respectively.

The fluorescent polarization assay was employed to conduct a screen to identify PptA inhibitors by using a small-molecule library comprising 6,231 diverse drug-like compounds (Table S3) at a concentration of 20 µM. The vast majority of these compounds (*n* = 6,117) had no noticeable effect (less than 10% inhibition) on PptA activity; however, we identified 66 compounds (1.1%) that inhibited activity by ≥50%. Three of these compounds, PD 404,182, 6-nitroso-1,2-benzopyrone, and calmidazolium chloride, have previously been shown to block the activity of the Sfp-type PPTase of the Gram-negative bacterial pathogen *Vibrio cholerae* (43). To assess the efficacy of these three compounds, we calculated the half-maximal inhibitory concentrations (IC_50_). All three compounds proved to be potent inhibitors of PptA, with IC_50_s of 3.9, 35.2, and 19.2 µM, respectively, for PD 404,182, 6-nitroso-1,2-benzopyrone, and calmidazolium chloride (Fig. 10). Interestingly, previously published data indicate that these compounds are inactive against the human orthologue AASDHPPT, suggesting the potential for the development of selective therapeutic inhibitors against PptA. This was confirmed for 6-nitroso-1,2-benzopyrone in an FP assay of the human phosphopantetheinyl transferase HS-PPTase, as no inhibition of the enzyme was observed at concentrations up to 200 µM.

10.1128/mBio.01504-16.7 TABLE S3  Results of high-throughput chemical library screening to identify AFpptA inhibitors (part A summarized the number of compounds identified, and part B reports MIC determinations for PptA inhibitors in the presence and absence of lysine and iron supplementation). Download TABLE S3, DOCX file, 0.01 MB. Copyright © 2017 Johns et al. 2017 Johns et al. This content is distributed under the terms of the Creative Commons Attribution 4.0 International license.

Compounds that have potent activity against an enzyme in an isolated assay often fail to translate this result to a biological effect; therefore, we evaluated the ability of PD 404,182, 6-nitroso-1,2-benzopyrone, and calmidazolium chloride to inhibit growth of *A. fumigatus* (CEA10) and *A. flavus* (NRRL 3357) in RPMI 1640 using a methodology based on the EUCAST standard broth microdilution assay. All three were relatively potent inhibitors of antifungal growth (Table S3). To confirm that the growth inhibition shown by these three compounds was related to inhibition of PptA and not other fungal PPTases (namely, PptB or the PPTase domain of FasA) or off-target effects, we attempted to reverse the action of drugs by supplementing medium with iron and lysine. The inhibitory effect of our control compound, amphotericin B, which acts to destabilize fungal membranes, was unaffected by supplementation; however, the antifungal activity of 6-nitroso-1,2-benzopyrone was reduced for both *A. fumigatus* (2-fold) and *A. flavus* (>4-fold).

Taken together these data suggest that PptA is both druggable and can be targeted in a selective way.

## DISCUSSION

Many of the antifungals used to treat invasive disease are blighted by significant pharmacological shortcomings which, combined with high rates of mortality associated with fungal disease and the emergence of resistance, particularly to the azoles, have stimulated the search for new antifungals with novel mechanisms of action (2, 4, 5). In this study, we have demonstrated the Sfp-PPTase PptA of *A. fumigatus* has the potential to be used as a therapeutic target.

Recently, the sole Sfp-type PPTase of *Mycobacterium tuberculosis*, PptT, was identified as a potential drug target for combating tuberculosis infection. PptT is required for the biosynthesis of mycobactins, mycolic acids, other polyketide-derived lipids, and siderophores and was found to be essential for viability and persistence in murine infection models (44). The role of the *A. fumigatus* Sfp-PPTase is somewhat similar to that of the *M. tuberculosis* enzyme. We have demonstrated that a *pptA* null mutant is unable to produce any detectable secondary metabolites, leading to, among other phenotypic defects, the loss of spore pigmentation and an inability to grow in iron-limiting environments. This is consistent with the proposed role of PptA in activation of the polyketide synthase PksP, which is in turn responsible for the first committed step in DHN-melanin biosynthesis and the reactions catalyzed by the NRPSs SidC and SidD, which are required for synthesis of the siderophores FC and TAFC, respectively. The role of PptA extends beyond that of the *Mycobacterium* PPTase in that it is required for the activation of the fungus specific enzyme l-α-aminoadipate reductase AarA, a key component in the lysine biosynthesis pathway (reference 26 and this study), and in keeping with this role, the *pptA* null mutant is unable to grow without supplemental lysine. Sfp-type PPTases have also been identified as essential virulence factors in a range of fungal plant pathogens (30, 45–47), but this is the first study to show this in a human fungal pathogen.

It has been suggested that antifungal targets should be sufficiently similar in a range of fungal pathogens to provide some confidence that any antifungal compound developed will have a broad spectrum of activity while still being sufficiently dissimilar from any host enzyme to allow selective targeting of the pathogen. There are three known classes of PPTases: type I or AcpS type, type II or Sfp type, and type III or Fas type. Our phylogenetic analysis of Sfp-type PPTases showed they are evolutionary distinct from the other types of PPTases. When examining the proteins found within this group, it is clear that there is reasonable conservation between AFPptA and those in many other pathogenic fungi, including *A. flavus* (64% identity) and the causative agents of coccidioidomycosis (*Coccidioides posadassi*, 55% identity) and histoplasmosis (*Histoplasma capsulatum*, 49% identity). The relationship between AFPptA and the orthologues in other significant pathogens is less compelling. No significant matches were found when we performed a BLASTp analysis with AFPptA against the genomes of *C. albicans* and *Cryptococcus neoformans*, and the orthologue ClustalW alignment scores are exceptionally low (6.38% for *C. albicans*, 14.02% for *C. neoformans*). AFPptA, however, is evolutionarily distant from the human Sfp-PPTase, aminoadipate-semialdehyde dehydrogenase–phosphopantetheinyl transferase (AASDHPPT), and shares low amino acid conservation (26% identity, 61% coverage). We conclude from our bioinformatics analyses that antifungals directed against PptA have the potential to be selective; however, they may be limited in their spectrum of activity.

The value of narrow-spectrum drug targets should not be discounted. Over the last 2 decades, significant progress has been made in the development of rapid diagnostic methods to identify the causative agent in fungal diseases, allowing clinicians to tailor their therapeutic approach. Treatment regimens already differ depending on the infective agent and site of infection, for example, the echinocandins are increasingly used for the treatment of disseminated candidiasis, but the azoles are preferred for the treatment of aspergillosis (4). Several clinicians and commercial bodies have also advocated the development of new antifungal classes that have activity restricted to either *Aspergillus* or *Candida* species (4, 48).

PptA has a role in the biosynthesis of numerous factors that have been directly associated with virulence, including lysine (27, 28), the siderophores FC and TAFC (7, 24), DHN-melanin (9, 49), and gliotoxin (15). This role, combined with our preliminary dose-response study results indicating that the *pptA* null mutant is unable to grow effectively at clinically relevant levels of lysine and iron, provides a highly plausible mechanistic basis for avirulence in mouse and larval models of *Aspergillus* infection. The distinct contributions of the aforementioned factors to *A. fumigatus* virulence do not appear to be equally weighted. Since virulence of *pksP* and *aarA* knockout mutants are indistinguishable from that of the isogenic parental isolates, we can surmise that the predominant factor promoting avirulence of the *pptA* null mutant in the larval model of infection is a reduced ability to sequester iron. This conclusion is further supported by our ability to rescue the virulence defect of the *pptA* null mutant by supplying iron during infection. In addition, a *sidA* null mutant, which lacks TAFC and FC biosynthetic capability, is similarly avirulent. Interestingly, our findings for *pksP* were somewhat in conflict with those of a previous study which indicated that an *A. fumigatus pksP* null mutant had increased virulence in a *Galleria mellonella* model compared to the wild type; however, this could reflect the different host strains used in the infection studies (50).

The loss of virulence of the Δ*pptA* strain in a murine infection model is likely to be multifactorial and complicated by the various challenges experienced by the mutant strain, depending on which organ is infected. Our study revealed that *pptA* is required for virulence in both bronchopulmonary and disseminated murine infection models. The role of lysine biosynthesis in virulence has been assessed in a number of studies. Strains lacking the ability to synthesize lysine are avirulent in bronchopulmonary but not disseminated models of infection (27, 28) and are thought to be able to overcome their deficiency in an invasive infection by liberating lysine via the degradative action of proteases on surrounding tissues. Targeting lysine biosynthesis alone is therefore not likely to resolve a preexisting infection. A previous study showed that a *sidA* null mutant was avirulent in a brochopulmonary infection model (7, 24); however, no data exist in relation to its ability to cause an infection in a disseminated model. The level of free iron found in human hosts is extremely low (10^−21^ mM to 10^−15^ mM), as the majority is found tightly bound to other molecules (34, 35). Thus, it is unlikely, given our results, that *A. fumigatus* will be able to overcome the loss of siderophores and thus *pptA* in an established infection.

The impact on virulence of the *pptA* null mutant may be further compounded by its lack of the ability to produce DHN-melanin. Not only have previous studies shown that DHN-melanin-deficient *A. fumigatus* isolates have reduced virulence (9, 49), but also melanin has been shown to play a key role in avoiding host detection and it has a protective effect against host killing (8–11, 39). Our data show that the *pptA* null mutant behaves in a similar manner as the *pksP* null mutant in its inability to prevent phagolysosome acidification. Interestingly, the *pptA* null mutant elicits an increased proinflammatory response when exposed to human dendritic cells that is significantly higher than the *pksP* null-elicited response. It has been shown that a lack of melanin leads to an irregular and hydrophilic coating of protein surrounding conidia (12). A difference in the consistency of this protein layer could be responsible for the diverging effects between the two DHN-melanin-deficient strains. Furthermore, this lack of secondary metabolites produced by the *pptA* null mutant could also be implicated. One such secondary metabolite that has been postulated to play a role in the host is gliotoxin.

Gliotoxin has been associated with the suppression of the adaptive immune response, the decrease of polymorphonuclear leukocyte-mediated inflammation, and the prevention of a respiratory burst in human polymorphonuclear leukocytes (13–15). Gliotoxin has also been postulated to play a role in fungal virulence (13, 14, 51–54). The first step of gliotoxin biogenesis requires the action of the NRPS GliP (51, 52). Although the lack of gliotoxin in *gliP* null isolates did not affect the virulence of *A. fumigatus* in neutropenic mice treated with cyclophosphamide and hydrocortisone (51, 52), attenuated virulence was observed in nonneutropenic mice immunosuppressed with corticosteroids (53, 54).

Taken together, the loss of these key virulence determinants in addition to the concomitant auxotrophies suggest that targeting PptA has the potential to provide an added level of protection above that possible by targeting any one of these critical virulence determinants individually.

The validation of a potential drug target is the first step in the development of a novel antifungal agent, yet without the ability to assess the activity of a target in a high-throughput assay, it is unlikely that compounds that can inhibit the function of the target will ever be found. In this study, we therefore developed a 384-well fluorescent polarization assay to monitor the activity of PptA. The assay is highly reproducible with a sufficient signal window to permit high-throughput screening. We calculated the Z′ factor, a parameter used to assess the suitability of high-throughput screens, to be 0.76. Typically, assays with Z′ values greater than or equal to 0.5 are considered suitable for HTS. We also determined that after stopping the reaction, the assay signal was stable for at least a week.

We used this assay to screen a compound library which included over 6,000 diverse small molecules from the MRCT index set (http://www.mrctechnology.org/researchers/), and we identified 66 compounds that inhibited PptA activity by >50% when used at a final concentration of 1 mM. Three of these compounds, PD 404,182, 6-nitroso-1,2-benzopyrone, and calmidazolium chloride, were assessed further and shown to be exceptionally potent inhibitors of *A. fumigatus* PptA. The effect of these compounds on the fungal enzyme is in stark contrast with their lack of detectable activity against the human orthologue AASDHPPT (43), indicating that selective agents could be developed. What is even more remarkable, given the lack of sequence conservation between fungal and bacterial enzymes, is that all three compounds are also potent inhibitors of *V. cholerae* Sfp-type PPTases (43). It is encouraging that all three have antifungal activity against both *A. fumigatus* and *A. flavus* (Table S3); however, the action of only one of these, 6-nitroso-1,2-benzopyrone, was partially reversed (2- to 4-fold) by supplementation of culture medium containing lysine and iron. This is consistent with the primary, but not only mechanism of antifungal activity of 6-nitroso-1,2-benzopyrone, being inhibition of PptA; moreover, these findings indicate that PptA is a druggable target.

In conclusion, PptA sits at a nexus, linking the biosynthesis of the siderophores TAFC and FC, lysine, DHN-melanin, gliotoxin, and all NRPS and PKS-derived secondary metabolites. Loss of AF*pptA* results in significant virulence defects in both bronchopulmonary and disseminated models of infection and an altered immune response. The lack of similarity between PptA and its human orthologue AASDHPPT, along with our ability to identify selective inhibitors and show that PptA is druggable, makes it an excellent antifungal drug target.

## MATERIALS AND METHODS

### Identification of sequences used in this study.

All *A. fumigatus* DNA sequences were downloaded from the Central Aspergillus REsource (CADRE) database (http://www.cadre-genomes.org.uk/index.html). The following sequences were used in this study: *pptA* (AFUB_024520A), *aarA* (AFUB_068270A), *sidA* (AFUB_023720A), and *pksP* (AFUB_033290A).

### Strains.

An A1160 Δ*Ku80 pyrG*^+^ strain, referred to as A1160p+ (55), served as the parental strain for all gene deletion mutant strains. The strains used are listed in Table 1. *A. fumigatus* was cultured on SAB agar at 37°C in the dark. Lysine auxotrophs were supplemented with 10 mM lysine. Strains auxotrophic for iron were supplemented with either 1.5 mM FeSO_4_ or 10 µM TAFC. TAFC was extracted from *A. fumigatus* in iron-deprived liquid cultures. Desferri-TAFC in the supernatant was saturated with iron and subsequently extracted using chloroform (56). *In vitro* susceptibility testing of mutant strains was carried out using the EUCAST broth microdilution reference method (57).

**TABLE 1:** Fungal strains used during this study

Strain^a^	Description	Source
A1160p+	Derived from CEA10; Ku80Δ *pyrG*^+^	55
Δ*pptA*	Derived from A1160p+, *hph*^+^ *pptA*^*−*^	This study
*pptA* rec	Reconstituted Δ*pptA*, *hph*^−^	This study
Δ*aarA*	Derived from A1160p+, *hph*^+^ *aarA*^*−*^	This study
*aarA* rec	Reconstitued Δ*aarA*, *hph*^−^	This study
Δ*pksP*	Derived from A1160p+, *hph*^+^ *pksP*^*−*^	This study
*pksP* rec	Reconstituted Δ*pksP hph*^*−*^	This study
Δ*sidA*	Derived from A1160p+, *hph*^+^ *sidA*^*−*^	This study
*Sida* rec	Reconstituted Δ*sidA*, *bleo*^+^ *sidA*^*−*^	This study
*A. flavus*	NRRL 3357	ARS Culture Collection

a All strains are *A. fumigatus* strains unless indicated otherwise.

### Generation of null mutants and reconstituted strains.

Gene knockout cassettes were generated using a modified PCR fusion approach (55, 58). All primers used to generate the cassettes can be found in Table S4. Primers P1 and P2 were used to amplify the 5′ noncoding regions and P3 and P4 were used to amplify the 3′ noncoding regions of the hygromycin B phosphotransferase gene (*hph*), which was amplified from pAN7-1 (59) using primers HPHF and HPHR. To facilitate gene fusion, primers P2 and P3 included regions complementary to the terminal regions of the *hph* primers. The PCR primer design software Primer 3 (http://frodo.wi.mit.edu/) was used to aid primer design, and all primers were supplied by Eurofins MWG Operon.

10.1128/mBio.01504-16.8 TABLE S4  Primers used in this study (part A lists primers for generation of *pptA*, *aarA*, *sidA*, and *pksP* gene knockout and reconstitution constructs, and primers for host response RT-PCR are listed in part B; part C lists primers for recombinant protein production). Download TABLE S4, DOCX file, 0.02 MB. Copyright © 2017 Johns et al. 2017 Johns et al. This content is distributed under the terms of the Creative Commons Attribution 4.0 International license.

*A. fumigatus* mycelia were treated with a 5% (wt/vol) Glucanex solution (made up in 0.6 M KCl–50 mM CaCl_2_ solution) for 2 to 3 h at 30°C to produce protoplasts. DNA was transformed into protoplasts by polyethylene glycol-mediated transformation. Hygromycin at 200 μg/ml was used for selection.

Reconstituted isolates *pptA* rec, *aarA* rec, *pksP* rec were generated by restoring the named genes at their original locus using fragments amplified with the associated primers P1 and P4. The *pptA* rec isolate was obtained by selection on Vogel’s minimal medium (VMM) (60) lacking supplemental lysine and iron, the *aarA* rec isolate was obtained on VMM lacking supplemental lysine, and the *pksP* rec isolate was identified as a green sporulating colony.

For the reconstituted isolate *sidA* rec, approximately 1 kb of 5′-flanking region in addition to the coding sequence and 3′-flanking region of genes of interest were amplified by PCR from fungal genomic DNA using LongAmp DNA polymerase. Primers P1 and P2 were used to amplify the 5′ region and the gene itself, and P3 and P4 were used to amplify the 3′-noncoding region of the gene (Table S4). The bleomycin resistance gene (*bleo*) was amplified from pPICZaA (Life Technologies, Inc.), incorporating regions complementary to P2 and P3 by using primers HPHF and HPHR. Reconstituted isolates were selected on VMM containing 40 µg/ml bleomycin.

All mutant and reconstituted strains were screened for homologous recombination by PCR and confirmed by Southern blotting (Fig. S4).

10.1128/mBio.01504-16.4 FIG S4  Southern blot analysis was used to confirm single integration of knockout (KO) and reconstitution cassettes in mutant strains. The restriction enzymes used for evaluation of each KO is shown along with the *in silico* prediction of restriction fragments that would be detected by the probe (gray box). (A) Evaluation of DNA isolated from the *pptA* null strain (Δ*pptA*), reconstituted pptA KI strain, and parental strain A1160. (B) Evaluation of DNA isolated from the *aarA* null mutant (Δ*aarA*) and control isolates. (C) Evaluation of DNA isolated from the *sidA* null mutant (Δ*sidA*) and control isolates. (D) Evaluation of DNA isolated from the *pksP* null mutant (Δ*pksP*) and control isolates. Bands present in all gene deletion and reconstitution strains show correct, single insertion of mutation cassettes. Download FIG S4, DOCX file, 0.7 MB. Copyright © 2017 Johns et al. 2017 Johns et al. This content is distributed under the terms of the Creative Commons Attribution 4.0 International license.

### Phylogenetic analysis.

Phylogenetic analyses were conducted using MEGA6 (61). All sequences were aligned using ClustalW software provided by MEGA6. Phylogenetic trees were prepared by the maximum likelihood method using the distance estimation Jones-Taylor-Thornton (JTT) model (62). Bootstrap values were calculated from 1,000 replications of the bootstrap procedure using programs within the MEGA6 package.

### Analyses of growth rates of null mutants.

RPMI 1640 with l-glutamine and NaHCO_3_ (Sigma) containing either 10 µM TAFC, 10 mM lysine, or both supplements was inoculated with 2.5 × 10^5^ spores/ml from the strains Δ*pptA*, *pptA*rec, and A1160p+. Two hundred microliters of the medium per spore mixture was transferred into a well of a 96-well plate, to give 5 × 10^4^ spores per well. Eight replicates of each strain were included in the plate, as well as 20 replicates of a negative control of medium alone. The plate was sealed with a Breathe Easy covering membrane (Sigma-Aldrich) and placed on a microplate scanning spectrophotometer (BioTek Synergy HT). The plate was incubated at 37°C, and the optical density at 600 nm was measured every 10 min over a 48-h time period. Fungal growth was determined by calculating the velocity of the linear phase of the growth curve. Student’s *t* test was used to determine the statistical significance between the growth rates of the different strains of *A. fumigatus*.

A dose-response assay was used to determine the optimum concentration of TAFC or lysine. For the TAFC dose-response assay, RPMI medium containing 10 mM lysine was used with 1:2 serial dilutions of TAFC from 10 µM. The lysine dose-response assay used RPMI medium containing 10 µM TAFC with 1:2 serial dilutions of lysine from 10 mM.

### Secondary metabolite assay.

A total of 1 × 10^7^ spores of strains Δ*pptA*, *pptA*rec, and A1160p+ were grown in 100 ml Czapek-dox medium or SAB medium for 7 days at 30°C. The culture supernatants were extracted with an equal volume of ethyl acetate. The organic phase was recovered and dried. The residuum was dissolved in 1 ml methanol and analyzed by LC-MS. Metabolites were identified by comparison with isolated standards (based on retention time and exact mass) as previously described (63).

### Response of dendritic cells to null mutants.

Peripheral blood mononuclear cells (PBMCs) were isolated from healthy donors by Ficoll-Hypaque density gradient centrifugation and cultured in growth medium (RPMI plus 1% [wt/vol] PenStrep plus l-glutamine and 10% [vol/vol] fetal bovine serum). DCs were obtained by separating monocytes via magnetic enrichment with anti-CD14 beads. DC differentiation occurred after 5 days of culturing in the presence of granulocyte-macrophage colony-stimulating factor (50 ng/ml) and recombinant IL-4 (25 ng/ml). Fresh medium was supplied on day 3.

*A. fumigatus* strains Δ*pptA*, *pptA* rec, Δ*pksP*, and A1160p+ were streaked onto SAB agar. After 3 days growth at 37°C, the spores were harvested through glass wool into phosphate-buffered saline (PBS). The spores were fixed by incubating in paraformaldehyde (2.5% [vol/vol]) overnight at 4°C. These were washed 3 times in PBS and suspended in growth medium. They were counted and normalized to the required concentration.

The DCs and *A. fumigatus* strains were coincubated for 24 h at a multiplicity of infection (MOI) of 2. After the required time RNA was extracted from the cells for quantitative real-time PCR (QRT-PCR) analysis. Total RNA was extracted from DCs by using a modified phenol and guanidine thiocyanate protocol (64).

A Brilliant II SYBR green QRT-PCR master mix one-step kit was used to synthesize cDNA and to perform PCR amplification using the primers listed in Table S4 (65). Transcripts for IL-1β and IL-6 genes were quantified using the Relative Expression software tool (REST) 2009 software. Quantification of the PCR signals was performed by comparing the cycle threshold (*C*_*T*_) value of the gene of interest with the *C*_*T*_ value of the reference gene, ACTβ. A two-sided Student’s *t* test was used for statistical analysis to compare wild-type responses to those of other strains. *P* values of <0.05 were considered significant.

### Phagolysosomal acidification assay.

*A. fumigatus* strains Δ*pptA*, *pptA* rec, Δ*pksP*, and A1160p+ were streaked onto aspergillus minimal media (AMM) (66) supplemented with 1.5 mM FeSO_4_ and 10 mM lysine, and after 5 days of growth at 37°C conidia were harvested.

Murine alveolar macrophages (ATCC TIB-71) were cultured in Dulbecco’s modified Eagle’s medium supplemented with 10% (vol/vol) fetal calf serum (Lonza), 1% (wt/vol) ultraglutamine (Lonza), and 27.5 µg/ml gentamicin (Lonza) at 37°C and 5% (vol/vol) CO_2_ to confluence.

RAW 264.7 macrophages were cultured overnight on microscopy coverslips in a 24-well plate. Prior to infection with conidia, the cells were prestained with 50 nM LysoTracker (Red DND-99; Thermo Fischer Science) for 1 h at 37°C in a CO_2_ incubator (10). Another aliquot of 50 nM LysoTracker was added with the infection of the macrophages. Fungal spores were stained with 100 µg/ml calcofluor white and administered at an MOI of 2. Plates were spun down for 5 min at 100 × *g*, 37°C, to synchronize phagocytosis. Cells were coincubated for 2 h at 37°C in a CO_2_ incubator.

Samples were washed once with PBS and fixed for 10 min at room temperature with 4% (vol/vol) formaldehyde. Cells were washed 3 times for 5 min with PBS, and coverslips were applied on glass slides for confocal laser scanning microscopy analysis.

Images were acquired with an inverted confocal laser scanning microscope (CFLSM; Carl Zeiss, Inc. AG) and analyzed using Zen software (Carl Zeiss, Inc. AG). Conidia surrounded by a ring-like LysoTracker signal were considered residing in an acidified phagolysosome and counted as acidified conidia. The ratio of acidified conidia to the total number of ingested conidia was determined to give the acidification ratio (10). All values represent mean results ± standard deviations (SD) of three experiments. Significance of the results was calculated with Student’s *t* test.

### *Galleria mellonella* infection model.

Sixth-stage instar larval *Galleria mellonella* moths (15 to 25 mm in length) were ordered from the Live Foods Company (Sheffield, England). Prior to use, the larvae were stored in the dark at 4°C in wood shavings. The larvae were used within 3 weeks of delivery. Randomly chosen groups of 30 *G. mellonella* organisms were inoculated by injecting 10 µl of a 1 × 10^6^-spores/ml suspension into the last, left proleg. This injection procedure ensured that the spores reached the hemocoel. Braun Omnican 50-U 100 0.5-ml insulin syringes with integrated needles were used. The needle was 12 mm in length and had a diameter of 0.3 mm. To ensure that the injection method or the original health status of the larvae was not responsible for any mortality observed, 3 control groups were included. These groups consisted of 30 larvae each; the first group was left untouched, the second was pierced with a needle, and the third group were injected with 10 µl phosphate-buffered saline with 0.05% (vol/vol) Tween 80 (PBS-T). Postinfection, the larvae were kept in petri dishes at 37°C in the dark, and survival was monitored daily for 7 days. Endpoints were characterized by a lack of movement or lack of response to stimulation and a black discoloration caused by melanization of the cuticle. Where stated, the larvae were treated with an iron source postinfection. Ten microliters of 1.5 mM FeSO_4_ was injected into the last, right proleg of the larvae immediately after initial fungal inoculation. The treatment continued once a day for the following 5 days. Each treatment was given into a different proleg. An additional control group of 30 larvae were included in the treatment study, in which larvae were injected with 1.5 mM FeSO_4_ once daily. After infection, the larvae were kept in petri dishes at 37°C in the dark, and survival was monitored daily for 5 days postinfection.

### Murine intranasal infection model.

The experiment was performed under UK Home Office project license PPL70/7324 and approved by the University of Manchester Ethics Committee. *A. fumigatus* was cultured on SAB agar at 37°C for 4 days before conidia were harvested by flooding with PBS-T. Viable counts from administered inocula were determined, following serial dilution, by growth for 24 to 48 h on SAB agar. Male CD1 mice weighing from 30 to 34 g (Charles River, Inc., Ltd.) were stored in groups of 5 in vented HEPA-filtered cages with access to food and water *ad libitum*. All mice were given 1 g/liter tetracycline hydrochloride (Sigma T8032) and 62.5 mg/liter ciprofloxacin (Pharmacy Ciproxin) in their drinking water throughout the course of the study. Mice were rendered leukopenic by administration of cyclophosphamide (150 mg/kg of body weight; intraperitoneal) on days −3, −1, +2, and every subsequent third day, and a single subcutaneous dose of hydrocortisone acetate (250 mg/kg) was administered on day −1.

Leukopenic male CD1 mice (25 to 30 g) were anesthetized by halothane inhalation and infected by intranasal instillation of spore suspensions of 5.0 × 10^5^ conidia/ml in 40 µl of PBS-T solution. Mice were weighed every 24 h from the day of first immunosuppression, and visual inspections were made twice daily. In the majority of cases, the endpoint for survival in experiments was sickness, at which point mice were sacrificed.

### Murine intravenous infection model.

The experiment was performed under UK Home Office project license PPL40/3101 and approved by the University of Manchester Ethics Committee. *A. fumigatus* was cultured on SAB agar at 37°C for 4 days before conidia were harvested by flooding with phosphate-buffered saline containing PBS-T. Viable counts from administered inocula were determined, following serial dilution, by growth for 24 to 48 h on SAB agar. Male CD1 mice weighing from 30 to 34 g (Charles River, Inc., Ltd.) were stored in groups of 5 in vented HEPA-filtered cages with access to food and water *ad libitum*. Mice were rendered neutropenic by a single dose of cyclophosphamide (200 mg/kg intraperitoneal) on day −3.

Neutropenic male CD1 mice (25 to 30 g) were infected by intravenous injection via lateral tail vein of spore suspensions of 6.5 × 10^5^ conidia/ml in 200 µl of PBS-T solution. Mice were weighed every 24 h from the day of first immunosuppression, and visual inspections were performed twice daily. In the majority of cases, the endpoint for survival was considered sickness, at which point mice were sacrificed.

### Cortisone-acetate-treated murine intranasal infection model.

Female CD1 mice weighing from 18 to 20 g (Charles River, Inc., Ltd.) were stored under standard conditions in individually ventilated cages with access to food and water *ad libitum*. All animals were cared for in accordance with the European animal welfare regulation and approved by the responsible federal/state authority and ethics committee in accordance with the German Animal Welfare Act (permit 03-001/12). Mice were immunosuppressed with two single doses of 25 mg cortisone acetate (Sigma-Aldrich), which were injected intraperitoneally 3 days before and immediately prior to infection with conidia (day 0). Mice were anesthetized by an intraperitoneal anesthetic combination of midazolam, fentanyl, and medetomidine and infected by intranasal instillation of spore suspensions of 2 × 10^5^ conidia/ml in 20 µl PBS. Anesthesia was terminated by subcutaneous injection of flumazenil, naloxon, and atipamezol. Infected animals were monitored twice daily to monitor for weight loss.

### Histological analysis.

For histological analysis, the lungs were fixed in buffered formalin and embedded in paraffin; 4-μm sections were stained using Periodic acid-Schiff stain.

GraphPad Prism was used to interpret all survival data. Kaplan-Meier survival analysis was used to create a population survival curve and to estimate survival over time, and *P* values were calculated through a log rank analysis (for comparative survival analysis).

### Preparation of recombinant PptA.

The complete PptA and AarA coding sequences were amplified from *A. fumigatus* cDNA by PCR using LongAmp DNA polymerase and the primers shown in Table S4. Purified products were cloned into pManHis vector (modified pET16b vector obtained from Eddie McKenzie, University of Manchester). Subsequently, PptA coding sequence was excised from pManHis by using the restriction enzyme BamH1 and subcloned into pGEX-6P-1 (GE Healthcare Life Sciences), a vector that contains an N-terminal GST tag designed to improve protein solubility. Human PPTase (HS-PPTase; also known as aminoadipate–semialdehyde dehydrogenase–phosphopantetheinyl transferase, or AASDHPPT) cDNA was amplified by PCR from Qiagen plasmid 5158986 using primers AASDHPPT_F and AASDHPPT_R and cloned into pET43.1 (Merck Millipore). A 264-bp fragment of the human fatty acid synthase cDNA was amplified by PCR from image clone 6172538 (Source Bioscience) using primers FASN_F and FASN_R and cloned into pET30. This fragment encodes amino acids 2118 to 2205 of the fatty acid synthase, known to be the acyl carrier protein domain (HS-ACP) that acts as a substrate for human PPTase. Cloning sites of plasmids were sequenced to ensure accurate amplification and correct insertion. *E. coli* BL21(DE3) cells were used for transformation of vectors. Expression was induced by the addition of isopropyl β-d-thiogalactoside at 0.5 mM. Cultures were then incubated for 16 h at 18°C. Cells were harvested and pelleted by centrifugation. For PptA and AarA, cell lysis was performed by sonication in lysis buffer (morpholineethanesulfonic acid [MES] at 50 mM [pH 6], NaCl at 500 mM, Tween 20 at 0.1% [vol/vol], phenylmethanesulfonyl fluoride at 1 mM, and lysozyme at 1 mg/ml). For HS-PPtase and HS-ACP *E. coli* was lysed using Bugbuster (Merck Millipore). Cell debris was removed by centrifugation at 16,000 × *g* for 20 min at 4°C.

AarA, HS-PPTase, and HS-ACP were purified using a Ni-nitrilotriacetic acid (Ni-NTA) His bind resin. AarA protein was eluted from the resin using 1 M imidazole in 50 mM MES (pH 6), 500 mM sodium chloride, and 0.1% (vol/vol) Tween 20. HS-PPTase and HS-ACP were eluted with 50 mM sodium phosphate (pH 8), 500 mM NaCl, 250 mM imidazole, 0.1% Tween 20. *pptA* cloned into pGEX-6P-1 was purified using glutathione-Sepharose resin affinity chromatography and was eluted by the addition of excess reduced glutathione (GST buffer kit; GE Healthcare Life Sciences). All purified proteins were desalted into assay buffer (62.5 mM bis-Tris, 12.5 mM MgCl; pH 6.5) with Sephadex G-25 columns (PD10; GE Healthcare Life Sciences).

### Assay for PptA activity.

The phosphopantetheinyl transferase activity of PptA was assessed in an FP assay. The assay monitored the transfer of the P-pant) from CoA to a target protein, AarA. The P-pant group of CoA was labeled with BODIPY*-*TMR iodoacetamide, as previously described (26). Titration of PptA (0 to 200 ng per reaction mixture) was performed with 750 ng AarA, 1 µl of BODIPY-TMR-labeled CoA, and assay buffer (62.5 mM bis-Tris, 12.5 mM MgCl; pH 6.5). This mixture was incubated at room temperature in the dark. The reaction was stopped at various time points by the addition of stop buffer (1 M EDTA; pH 8). FP was measured in a Synergy 2 microplate reader (excitation, 530/25; emission 590/35; gain, 1,000). Results were plotted onto a scatter graph using Microsoft Excel software. Repeatability of the assay over the 384-well plate was determined by mixing 100 ng PptA with 750 ng AarA, 1 µl of BODIPY-TMR-labeled CoA, and assay buffer (62.5 mM bis-Tris, 12.5 mM MgCl; pH 6.5) in the presence or absence of 1 M EDTA. This mixture was incubated at room temperature in the dark for 30 min. Data were plotted in an aligned scatter graph using Microsoft Excel software, and the Z′ value was calculated following procedures laid out by Brooks et al. (67). For fluorescence polarization assay using the human proteins, 400 ng of HS-PPTase and 1,500 ng of HS-ACP were assayed under the same reaction conditions.

Inhibitor screens were performed using the 384-well plate-based assay described above with the aid of a Biomek FX robotic system using compounds at a final concentration of 1 mM (in 1% dimethyl sulfoxide. IC_50_s were determined in triplicate using doubling dilutions of compounds from 100 µM. IC_50_ calculations were performed using GraphPad Prism 6.
