# Supplementary material for: A Nonredundant Phosphopantetheinyl Transferase, PptA, Is a Novel Antifungal Target That Directs Secondary Metabolite, Siderophore, and Lysine Biosynthesis in Aspergillus fumigatus and Is Critical for Pathogenicity
Source: mBio. 2017 Jul 18;8(4):e01504-16. doi: 10.1128/mBio.01504-16 (PMC5516258; doi:10.1128/mBio.01504-16)
Supplement: FIG S1 [file mbo003173360sf1.docx]

 **Fig S1: Phosphopantetheinylation.** The 4’-phosphopantetheine (P-pant) group within Coenzyme A is transferred to a conserved serine residue in a peptidyl carrier domain of an inactive apo-carrier protein to create an active holo-carrier protein. This process is facilitated by 4′-phosphopantetheinyl transferase (4’-PPTase) (adapted from (6)).
