# Supplementary material for: A Nonredundant Phosphopantetheinyl Transferase, PptA, Is a Novel Antifungal Target That Directs Secondary Metabolite, Siderophore, and Lysine Biosynthesis in Aspergillus fumigatus and Is Critical for Pathogenicity
Source: mBio. 2017 Jul 18;8(4):e01504-16. doi: 10.1128/mBio.01504-16 (PMC5516258; doi:10.1128/mBio.01504-16)
Supplement: TABLE S1 [file mbo003173360st1.docx]

Table S1: Predicted and detected masses of TAFC identified in culture supernatants from the isogenic parent (wt) and the pptA reconstituted strain pptArec

|  | exact mass |
| --- | --- |
| TAFC calculated positive mode | 906.3309 |
| TAFC calculated negative mode | 904.3153 |
| Desferri-TAFC calculated positive mode | 853.4194 |
| Desferri-TAFC calculated negative mode | 851.4038 |
| isolated TAFC positive mode detected | 906.4910 |
| isolated TAFC negative mode detected | 904.3699 |
| isolated Desferri-TAFC negative mode | 851.4351 |
| wt TAFC positive mode | 906.4545 |
| wt Desferri-TAFC negative mode | 851.4677 |
| pptArec strain TAFC positive mode | 906.5704 |
