# Supplementary material for: A Nonredundant Phosphopantetheinyl Transferase, PptA, Is a Novel Antifungal Target That Directs Secondary Metabolite, Siderophore, and Lysine Biosynthesis in Aspergillus fumigatus and Is Critical for Pathogenicity
Source: mBio. 2017 Jul 18;8(4):e01504-16. doi: 10.1128/mBio.01504-16 (PMC5516258; doi:10.1128/mBio.01504-16)
Supplement: TABLE S2 [file mbo003173360st2.docx]

Table S2: List of ClustalW pairwise alignment scores and BlastP analysis against *A. fumigatus* PptA. N/A represents no significant similarity found. Shading represents ClustalW pairwise alignment scores > 50%.

| **Organism** | **Gene Name** | **Sequence length** | **ClustalW alignment score** | **BlastP** | |
| --- | --- | --- | --- | --- | --- |
|  |  |  |  | **Identity** | **Query Cover** |
| *Aspergillus clavatus* | *npgA* | 346 | 73.12 | 72 | 99 |
| *Aspergillus flavus* | *npgA* | 343 | 66.76 | 64 | 99 |
| *Aspergillus terreus* | *npgA* | 324 | 60.8 | 56 | 99 |
| *Coccidioides immitis* | *npgA* | 328 | 57.32 | 56 | 96 |
| *Coccidioides posadasii* | *npgA* | 334 | 56.59 | 55 | 96 |
| *Blastomyces dematitidis* | *npgA* | 355 | 52.11 | 53 | 95 |
| *Aspergillus niger* | *npgA* | 308 | 53.9 | 51 | 96 |
| *Paracoccidioides lutzii* | *npgA* | 328 | 47.87 | 48 | 96 |
| *Arthroderma benhamiae* | *npgA* | 311 | 53.7 | 50 | 96 |
| *Trichophyton rubrum* | *npgA* | 313 | 52.72 | 49 | 96 |
| *Arthroderma otae* | *npgA* | 284 | 50.0 | 47 | 84 |
| *Microsporum gypseum* | *npgA* | 311 | 50.8 | 47 | 96 |
| *Histoplasma capsulatum* | *npgA* | 311 | 50.8 | 49 | 94 |
| *Saccharomyces cerevisiae* | *lys5* | 272 | 14.71 | 31 | 24 |
| *Cryptococcus neoformans* | CNAG_03174 | 264 | 14.02 | N/A | N/A |
| *Magnaporthe oryzae* | MGG_17878 | 325 | 32.92 | 32 | 87 |
| *Candida albicans* | *lys5* | 329 | 6.38 | N/A | N/A |
| *Mycobacterium tuberculosis* | *pptT* | 227 | 5.29 | N/A | N/A |
| *Bacillus subtilis* | *ppt1* | 224 | 16.96 | N/A | N/A |
| *Escherichia coli* | *entD* | 256 | 1.56 | N/A | N/A |
| *Homo sapiens* | AASDHPPT | 309 | 21.04 | 26 | 61 |
| *Mus musculus* | AASDHPPT | 309 | 17.8 | 26 | 62 |
