# Supplementary material for: A Nonredundant Phosphopantetheinyl Transferase, PptA, Is a Novel Antifungal Target That Directs Secondary Metabolite, Siderophore, and Lysine Biosynthesis in Aspergillus fumigatus and Is Critical for Pathogenicity
Source: mBio. 2017 Jul 18;8(4):e01504-16. doi: 10.1128/mBio.01504-16 (PMC5516258; doi:10.1128/mBio.01504-16)
Supplement: FIG S2 [file mbo003173360sf2.docx]

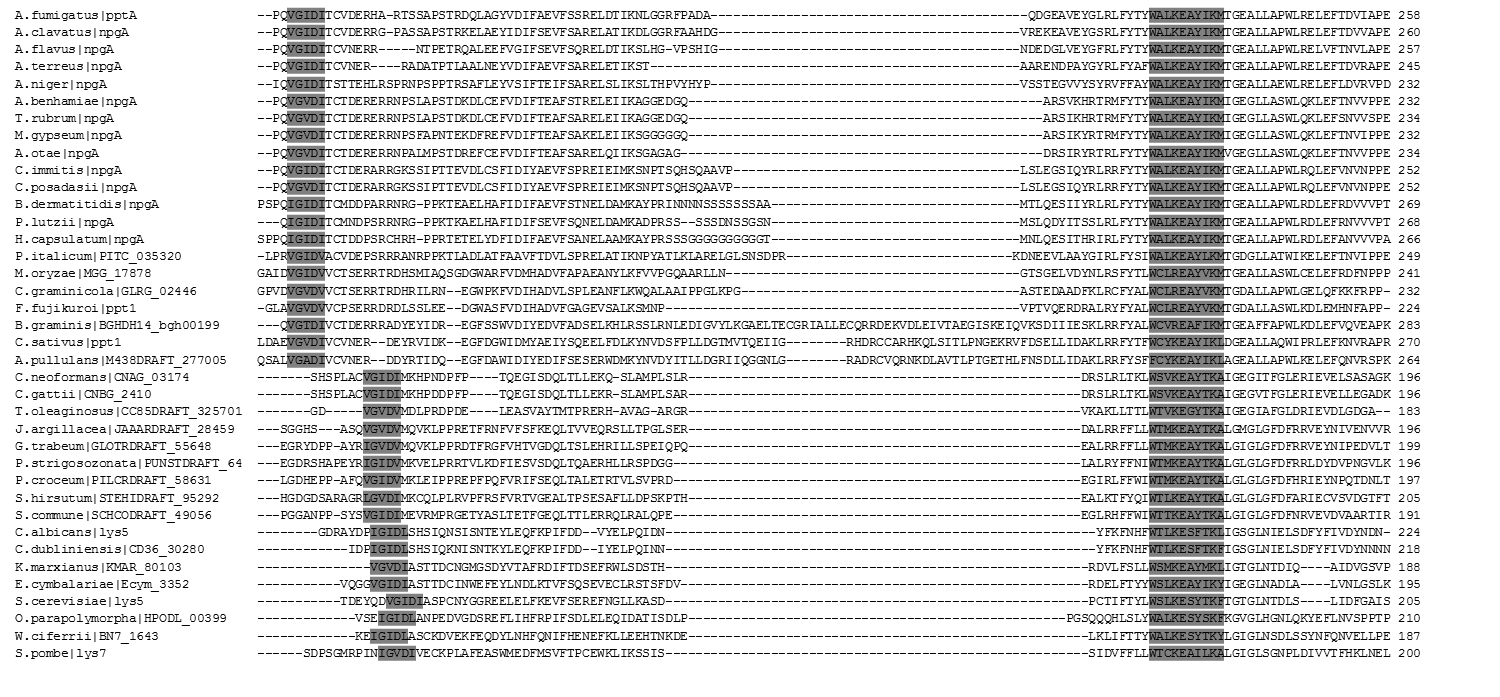
**Fig S2: Fungal Sfp-PPTases signature.** Fungal Sfp-PPTases can be identified based on the modified signature (I/V/L)G(I/V/A/T)D(I/V/L)(x)n(F/W)(A/S/T/C)x(K/R)E(S/A)h(h/S)K(M/L/A/F) where n is 41-107 aa. Grey shading indicates conserved signature.
