# Supplementary material for: A Nonredundant Phosphopantetheinyl Transferase, PptA, Is a Novel Antifungal Target That Directs Secondary Metabolite, Siderophore, and Lysine Biosynthesis in Aspergillus fumigatus and Is Critical for Pathogenicity
Source: mBio. 2017 Jul 18;8(4):e01504-16. doi: 10.1128/mBio.01504-16 (PMC5516258; doi:10.1128/mBio.01504-16)
Supplement: FIG S3 [file mbo003173360sf3.docx]

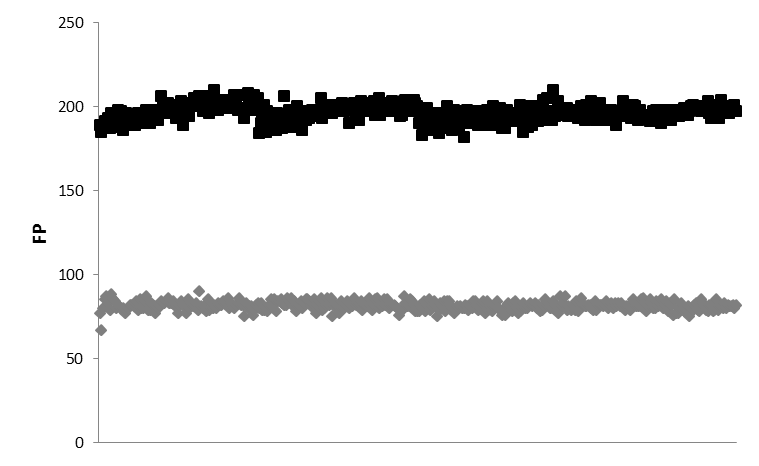


Z’=0.76

Fig S3: Repeatability determination of AFPptA assay over 384 well plate. Each point represents the FP value obtained from the AFpptA assay in the presence (black) or absence (grey) of the metal chelator and assay inhibitor EDTA.
