# Supplementary material for: A Nonredundant Phosphopantetheinyl Transferase, PptA, Is a Novel Antifungal Target That Directs Secondary Metabolite, Siderophore, and Lysine Biosynthesis in Aspergillus fumigatus and Is Critical for Pathogenicity
Source: mBio. 2017 Jul 18;8(4):e01504-16. doi: 10.1128/mBio.01504-16 (PMC5516258; doi:10.1128/mBio.01504-16)
Supplement: TABLE S3 [file mbo003173360st3.docx]

# Table S3 Results of high throughput chemical library screen to identify AFpptA inhibitors. A: Number of compounds identified. B: MIC determination of PptA inhibitors in the presence and absence of lysine and iron supplementation

# A

|  | Percentage inhibition of PptA activity | | | | | |
| --- | --- | --- | --- | --- | --- | --- |
|  | < 50 % | 50-60% | 60-70% | 70-80% | 80-90% | >90% |
| Number of compounds | 6165 | 20 | 17 | 11 | 3 | 15 |

# B

|  | MIC (mg/L) | | | |
| --- | --- | --- | --- | --- |
|  | A. fumigatus (A1160) | | A. flavus (NRRL 3357) | |
|  | RPMI | RMPI+lysine+iron | RPMI | RMPI+lysine+iron |
| 6- Nitroso-1,2-Benzopyrone | 10 | 20 | 10 | >20 |
| PD 404,182 | 6.25 | 6.25 | 12.5 | 12.5 |
| calmidazolium chloride | 0.31 | 0.31 | 0.31 | 0.31 |
| Amphotericin B | 0.25 | 0.25 | 0.5 | 0.5 |
