# Supplementary material for: A Nonredundant Phosphopantetheinyl Transferase, PptA, Is a Novel Antifungal Target That Directs Secondary Metabolite, Siderophore, and Lysine Biosynthesis in Aspergillus fumigatus and Is Critical for Pathogenicity
Source: mBio. 2017 Jul 18;8(4):e01504-16. doi: 10.1128/mBio.01504-16 (PMC5516258; doi:10.1128/mBio.01504-16)
Supplement: TABLE S4 [file mbo003173360st4.docx]

Table S4: Primers used in this study

| **Gene** | **Application** | **Primer** | **Sequence** |
| --- | --- | --- | --- |
| ***hph*/*ble*** | **Application** | HPHF | CCGGCTCGGTAACAGAACTAACGGCGTAACCAAAAGTCAC |
|  |  | HPHR | GGGAGCATATCGTTCAGAGCTCTTGACGACCGTTGATCTG |
| ***pptA*** | **2 step fusion PCR** | pptA_P1 | CCGGTCTCTTTCTCTGCATC |
|  |  | pptA_P2 | TAGTTCTGTTACCGAGCCGGTCAAACGAGGGAGGAGTCAG |
|  |  | pptA_P3 | GCTCTGAACGATATGCTCCCCCATGCAATATTCCACAGGA |
|  |  | pptA_P4 | CGGCGTACAGTTCGACATTA |
|  |  | pptA_P5 | CGTCCACCTGGATACCTTGT |
|  |  | pptA_P6 | TCTTCATTGGCAACCATCAG |
|  | **Amplification Validation** | pptAF | ACCACCTCAGGGACAGACAC |
|  |  | pptAR | CTCCTTGAGAGCCCAGTACG |
| ***aarA*** | **2 step fusion PCR** | aara_P1 | ATCACCGTGAATGGATTCGT |
|  |  | aara_P2 | TAGTTCTGTTACCGAGCCGGAACTCTTGTTTGGGCACTCG |
|  |  | aara_P3 | GCTCTGAACGATATGCTCCCGCGATTTTCAGAGACACACG |
|  |  | aara_P4 | TTAAGCTGACGGCAGAGGTT |
|  |  | aara_P5 | TCATCGAGAGTGATGGACGA |
|  |  | aara_P6 | CGCGGTAACAGCCTCATTAT |
|  | **Amplification Validation** | aarAF | TTCATCACGCTTTCTTCGTG |
|  |  | aarAR | TCAGCACGTCCAGAACACTC |
| ***sidA*** | **2 step fusion PCR** | sidA­_P1 | CCTGCTCCAGGGTAGGTGTA |
|  |  | sidA­_P2 | TAGTTCTGTTACCGAGCCGGAGAGATGTGGGAGGAGCAG |
|  |  | sidA­_P3 | GCTCTGAACGATATGCTCCCGCCATTTCTCTGACAACACG |
|  |  | sidA­_P4 | GCCGTCTGTTACATCCAGGT |
|  |  | sidA­_P5 | CAGATGAGATGGGCACACTG |
|  |  | sidA­_P6 | AGCAATTGTCTGTGCAAACG |
|  | **Amplification Validation** | sidAF | TTCTGTGTGTTGGGTTTGGA |
|  |  | sidAR | CCGATTGCAATAACGACCTT |
|  | **Reconstituted strain** | sidArec_P1 | CCTGCTCCAGGGTAGGTGTA |
|  |  | sidArec_P2 | TAGTTCTGTTACCGAGCCGGTTGACTGCGCCAGAATAGT |
|  |  | sidArec_P3 | GCTCTGAACGATATGCTCCCCGTTGTAAAGCTTTCATGGAG |
|  |  | sidArec_P4 | CGATGTAGTAAAGCGGCAAC |
| ***pksP*** | **2 step fusion PCR** | pksP­_P1 | CACCCTCATAACGACCCAAC |
|  |  | pksP­_P2 | TAGTTCTGTTACCGAGCCGGGCCGTGACTGCAAGGAGTAG |
|  |  | pksP­_P3 | GCTCTGAACGATATGCTCCCTTGTTCACAGCCTTGGTCAG |
|  |  | pksP­_P4 | TCACATCCAGCAATGTGTCA |
|  |  | pksP­_P5 | ATGCAACATGCAACCCTACA |
|  |  | pksP­_P6 | TGTCTCATCTTCCCCTCAGC |
|  | **Amplification Validation** | pksPF | GACCGTACAGCTTTGGTGGT |
|  |  | pksPR | GACAACTCCTTCGCCTTCTG |

# A: Primers for generation of *pptA*, *aarA*, *sidA* and *pksP* gene knock out and reconstitution constructs

B: Primers for host response RT-PCR

| **Gene** | **Primer** | **Sequence** |
| --- | --- | --- |
| **IL-1β** | IL1β_F | GCTGATGGCCCTAAACAGATG |
|  | IL1β_R | TAGTGGTGGTCGGAGATTCGT |
| **IL-6** | IL6_F | ACTCACCTCTTCAGAACG |
|  | IL6_R | GGCTTGTTCCTCACTACT |
| **ACTβ** | ACTβ_F | GTGATGGTGGGCATGGGTC |
|  | ACTβ_R | ACACGCAGCTCATTGTA |

C: Primers for recombinant protein production

| **Protein** | **Primer** | **Sequence** |
| --- | --- | --- |
| **PptA** | PptA_F | TATGGATCCGCACAAAACGAGAGGATACC |
|  | PptA _R_pManHis | TATGGATCCCTACTAGGGCTGTTTTTTTGTACACTGACAGA |
|  | PptA _R_pGEX | TATGGATCCCTACTAATGATGATGATGATGATGGGGCTGTTTTTTTGTACACTGACAGA |
| **AarA** | AarA_F | TATGGATCCGGTGTTGAAACAGCCTCCTT |
|  | AarA _R | ATGAATTCCTACTAAGAAGTGCCCCCACGTCCAC |
