# Supplementary material for: A Nonredundant Phosphopantetheinyl Transferase, PptA, Is a Novel Antifungal Target That Directs Secondary Metabolite, Siderophore, and Lysine Biosynthesis in Aspergillus fumigatus and Is Critical for Pathogenicity
Source: mBio. 2017 Jul 18;8(4):e01504-16. doi: 10.1128/mBio.01504-16 (PMC5516258; doi:10.1128/mBio.01504-16)
Supplement: FIG S4 [file mbo003173360sf4.docx]

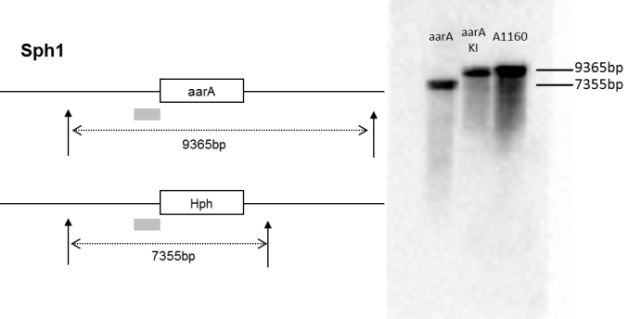


**B**


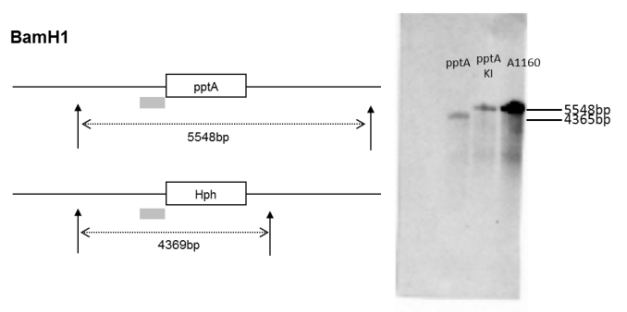


**A**


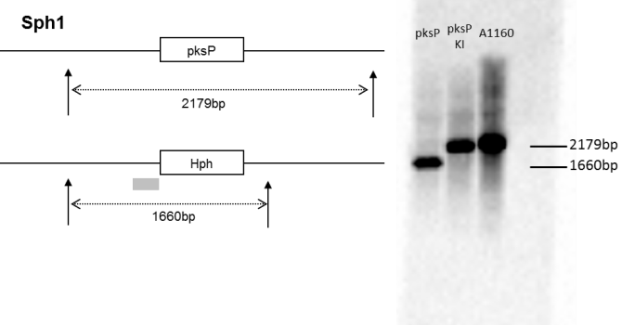


**D**


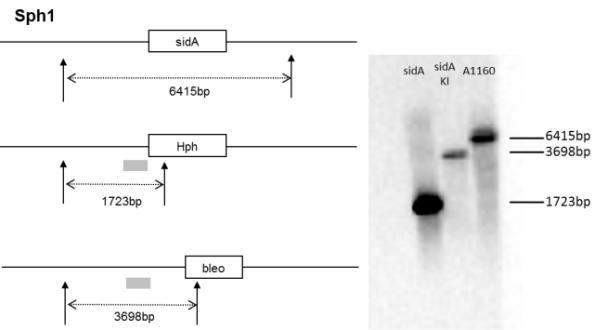


**C**

**Fig S4: Southern blot analysis was used to confirm single integration of knock out and reconstitution cassettes in mutant strains.** The restriction enzymes used for evaluation of each KO is shown along with the *in silico* prediction of restriction fragments that would be detected by the probe (grey box). A) Evaluation of DNA isolated from the *pptA* null (∆*pptA*) reconstituted (*pptA* KI) and parental (A1160). B) Evaluation of DNA isolated from the *aarA* null (Δ*aarA*) and control isolates. C) Evaluation of DNA isolated from the *sidA* null (Δ*sidA*) and control isolates. D) Evaluation of DNA isolated from the *pksP* null (Δ*pksP*) and control isolates. Bands present in all gene deletion and reconstitution strains show correct, single insertion of mutation cassettes.
